# Structural and Compositional Effects on the Scintillation Properties of Fast Emitting Metal‐Organic Frameworks

**DOI:** 10.1002/advs.75224

**Published:** 2026-04-16

**Authors:** Francesca Cova, Jacopo Perego, Biplab Joarder, Nobuhiro Yanai, Anna Vedda, Silvia Bracco, Angiolina Comotti, Angelo Monguzzi, Irene Villa

**Affiliations:** ^1^ Department of Materials Science University of Milano – Bicocca Milano Italy; ^2^ Advanced Membranes & Porous Materials Platform (AMPMP) Division of Physical Sciences and Engineering King Abdullah University of Science and Technology Thuwal Saudi Arabia; ^3^ Department of Chemistry Graduate School of Science The University of Tokyo Tokyo Japan; ^4^ NANOMIB BioNanoMedicine Center University of Milano – Bicocca Milano Italy

**Keywords:** energy diffusion, fast emitters, hybrid materials, metal organic frameworks, molecular excitons, scintillation

## Abstract

Metal‐organic framework (MOF) scintillators are promising emitters for fast timing techniques due to their high scintillation efficiency and rapid response time. These materials are composed of high‐atomic‐number metal‐oxide cores connected by scintillating organic dyes. A quite‐overlooked drawback of MOF scintillation properties is the possible presence of intrinsic structural defects and dark states that can introduce energetic disorder and also trap the energy released by the ionizing radiation. Here, we systematically investigate the luminescence properties of three different MOF architectures wherein the scintillating ligand is 9,10‐diphenylanthracene and the linking nodes consist of Zr, In and Hf‐oxo‐hydroxy clusters. The various crystalline structures, the atomic numbers of the constituent elements, and the size of MOF crystals affect their photoluminescence and scintillation properties in distinct ways, providing guidelines to develop systems with optimized performance and tailorable timing capabilities, resulting from the synergy among the properties of the components.

## Graphical abstract

The crystalline structure, the atomic numbers of the constituent elementsand the size of MOF crystals each influence their photoluminescence and scintillation properties in distinct ways. Together, these factors provide valuable guidelines for designing systems with optimized performance and tunable timing characteristics, arising from the synergistic interplay of their components.

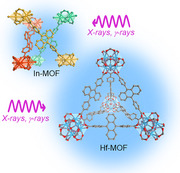

## 1 Introduction

Scintillators are materials able to down‐convert the high energy of ionizing radiation into UV/visible light [1] through several mechanisms, namely the photoelectric effect, the Compton effect and pairs production, whose probability and yield depend on the atomic number *Z* and on the density of the material, as well as the energy of the radiation [2]. A plethora of inorganic halide scintillators together with oxides and garnets have been used for gammarays spectroscopy, neutron detection, homeland security, geological exploration, as well as for high‐energy physics and medical imaging [3, 4, 5, 6, 7, 8]. These materials possess the high *Z* and high density required for an efficient photoelectric interaction, with high intrinsic light yield (LY, number of emitted photons over unit of absorbed energy) usually larger than 10^4^ ph MeV^−1^, enabling to fabricate detectors with good energy resolution [9, 10]. However, they are often slow emitters, with scintillation kinetics that spans from tens of nanoseconds to microseconds. This prevents their use in advanced fast timing techniques like time‐of‐flight positron emission tomography for medical imaging or high‐event rate high‐energy physics experiments where always higher detection rates and sensitivity are required [11, 12]. On the other hand, since the early 1980s the category of scintillators belonging to organic conjugated systems has been promising for the detection of particles and fast neutrons. Characterized by nanosecond‐scale timing properties and good LY values typically in the order of 10^3^–10^4 ^ph MeV^−1^, they are potentially exploitable for fast‐timing, but being composed of low‐Z elements such as carbon and hydrogen, their stopping power is low, with a consequent weak interaction with the high energy radiation and therefore a low light output [13, 14, 15, 16].

Recently, the use of composite materials made of inorganic nanoparticles or hybrid materials, such as perovskites, embedded in polymers and organic solvents or coupled to organic dyes, has been evaluated to create scintillators with emission properties and dynamics that can be tailored according to the foreseen application by controlling their size, morphology, surface quality, and composition, enabling to address the demands of technological advances in expertise fields where the traditional materials fail [17, 18, 19, 20, 21, 22, 23, 24, 25, 26, 27, 28, 29, 30, 31, 32, 33, 34, 35, 36]. In these hybrid systems, the high‐Z moieties could increase the average density and interact more efficiently with the high energy photons and the generated secondary charges, thus partially compensating for the poor stopping power of organic materials [37, 38, 39]. In this context, nanoporous and luminescent MOFs composed of inorganic metal‐oxide clusters as bridging units for organic ligands [40, 41, 42, 43, 44, 45, 46, 47], can be considered as a new archetypal example of hybrid crystalline scintillators. They have recently become attractive as scintillators due to their excellent luminescence and compositional versatility that allow to tailor their properties with a good control over structural stability, scintillation speed and LY [40, 48, 49, 50, 51, 52, 53, 54, 55, 56]. For instance, we demonstrated that selecting ligands with complementary electronic properties enables to tune the MOF performances in terms of emission Stokes shift. This happens through the exploitation of ultrafast energy transfer processes within the ligand framework, which allows to eliminate self‐absorption [52] and improves the LY [54] while preserving the fast time response therefore overcoming some of the issues that afflict other materials such as perovskites, organic and more traditional inorganic scintillators [57].

**FIGURE 1:**
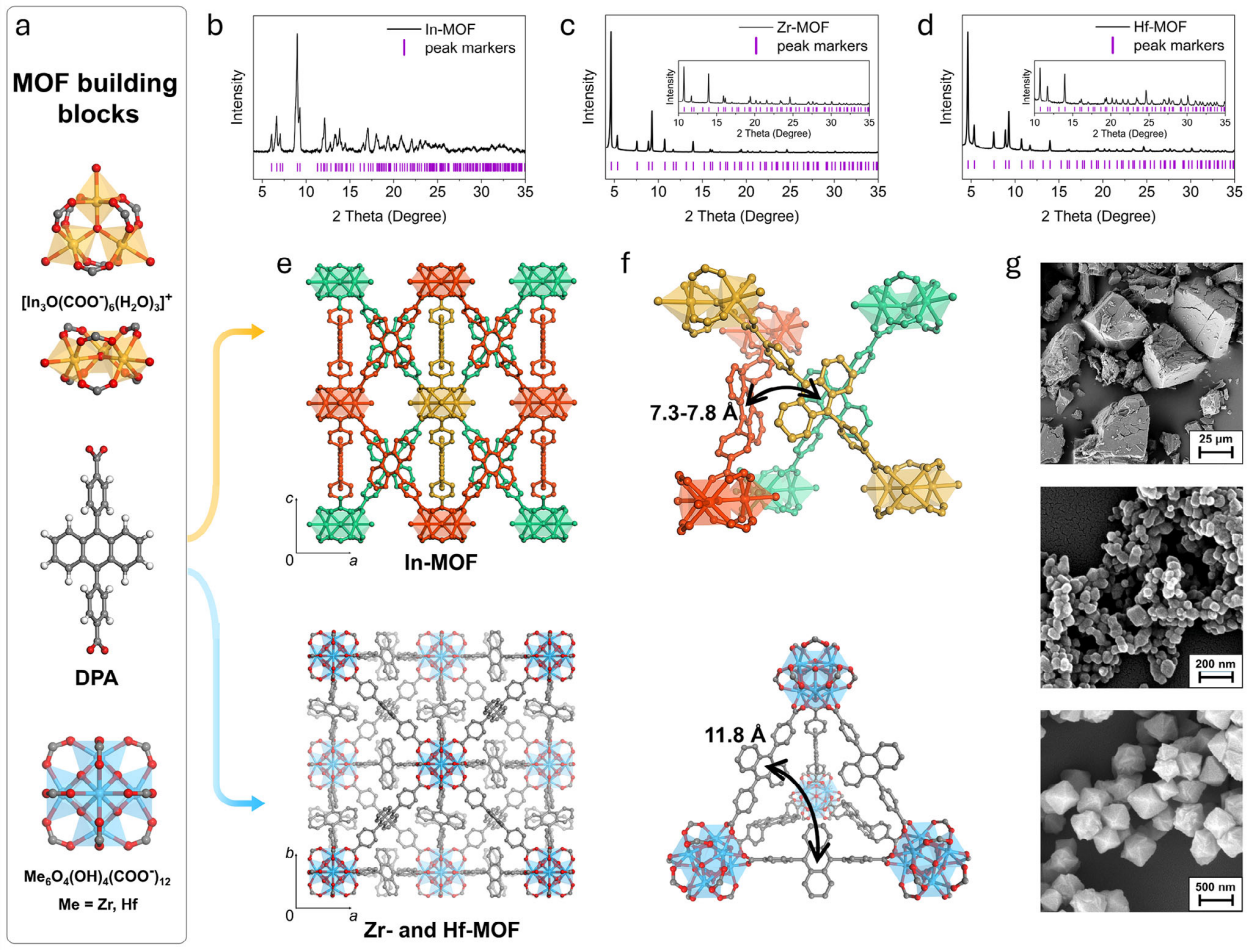
(a) Molecular structure of MOF building blocks, respectively the In‐oxo‐hydroxy cluster (top), the fluorescent ligand DPA (middle), and the Hf‐, Zr‐oxo‐hydroxy cluster (bottom). Powder X‐ray diffraction patterns of (b) In‐MOF, (c) Zr‐MOF, and (d) Hf‐MOF. The purple markers show the positions of the reflections calculated for In‐MOF, and Zr‐/Hf‐MOF from Pawley and Rietveld refinement, respectively. (e) Crystal structures of the luminescent In‐MOF in the *ac*‐plane (top) and of Zr‐ and Hf‐MOFs in the *ab*‐plane (bottom). The Cl^−^ anions and some of the DPA ligands in the In‐MOF, and the disorder in the Zr‐ and Hf‐MOF were removed for clarity. The different sub‐networks of the In‐MOF are highlighted in yellow, red, and green, respectively. (f) The center‐to‐center distances of nearest neighbor ligands in the In‐MOF structure (top) and in the Zr‐ and Hf‐MOF structures (bottom) (g) SEM images of the In‐MOF (top), Zr‐MOF (middle), and Hf‐MOF (bottom).

Notably, for any given ligand, a large variety of scintillating MOF topologies can be assembled using highly connected inorganic clusters with precise coordination geometry [58]. Therefore, in the design of novel MOFs the final material performance will be far from being a trivial sum of properties of its components, since even a small change in the architecture and composition can lead to a different behavior. In this regard, while a considerable amount of research has been devoted to the study of the structure, pore dimensions, compositions and flexibility of MOFs with respect to their applications in gas storage and separation, water harvesting, and catalysis [56, 59, 60], the understanding of the composition‐structure‐scintillation relationship for these materials is still in its infancy. Here, we investigate three MOFs based on the same scintillating conjugated ligand 4,4′‐(anthracene‐9,10‐diyl)dibenzoate (DPA) and three distinct metal‐oxide linking nodes containing different metal ions, Zr, Hf and In (Figure 1a). Specifically, we investigated and compared the scintillation properties of Zr‐ and Hf‐MOFs, which share the crystal structure but different atomic numbers (*Z*_Zr_ = 40 and *Z*_Hf_ = 72, respectively) [52, 53], whilst in In‐MOF the ligands are framed in a different architecture although the *Z*_In_ = 49 is comparable to that of Zr. Photoluminescence, scintillation and thermally stimulated luminescence experiments point out the structure/composition‐related key factors affecting the MOF luminescence and time response, shedding new awareness on the way to design systems with properties ad hoc according to the target applications.

## 2 Results and Discussion

### 2.1 Synthesis and Structural Investigation of MOFs

Figure 1a shows the molecular structure of the scintillating ligand 4,4′‐(anthracene‐9,10‐diyl)dibenzoate (DPA) and of the metal‐oxo‐hydroxy clusters used as linking nodes. In‐MOF was synthesized via a solvothermal reaction between DPA and InCl_3_ in a mixed solvent system at 120°C over a period of 3 d. Single‐crystal X‐ray diffraction analysis revealed that In‐MOF crystallizes in the orthorhombic crystal system with the *Cmcm* space group, and its chemical formula is assigned as In_3_(C_28_H_16_O_4_)_3_O(H_2_O)_3_Cl]_n_·solvents. Each In^3^^+^ center adopts an octahedral coordination geometry, being coordinated to four carboxylate oxygen atoms from four different DPA ligands, one oxygen atom from a µ_3_‐oxo bridge, and one oxygen atom from a coordinated water molecule (Figure 1a). The high free volume of this lightweight structure promotes the generation of a triply interpenetrated architecture. The phase purity of the resulting material was confirmed by powder X‐ray diffraction and Pawley refinement (Figure 1b, Figure S1, and Table S1). Zr‐MOF and Hf‐MOF nanocrystals were prepared using a solvothermal synthesis method in the presence of DPA building blocks and ZrCl_4_ and HfCl_4_, respectively [52]. Acetic acid and formic acid, respectively, control the crystallization process and promote the formation of highly crystalline nanoparticles (Figure 1c,d). The DPA ligands are coordinated to Zr (IV) and Hf (IV) ions, creating isostructural Zr‐MOF and Hf‐MOF with a **fcu** topology and a face‐centered cubic crystal structure (*Fm*‐3*m* space group, Figure 1e), as obtained by Rietveld refinement of PXRD patterns (Figures S2 and S3 and Table S2). Each Zr‐ or Hf‐based node (Me_6_(µ_3_‐O)_4_(µ_3_‐OH)_4_(CO_2_)_12_ cluster) is coordinated to 12 ligands, yielding a framework containing interconnected octahedral and tetrahedral cavities. The connectivity, purity, and thermal stability were demonstrated by FT‐IR, ^1^H solution NMR, and TGA analysis (Figures S5–S9).

Notably, the center‐to‐center intermolecular distances between neighbor ligands were tuned by the crystal frameworks: the intermolecular distance is 11.8 Å for the Zr‐MOF and Hf‐MOF, whilst it shortens to 7.3 Å for In‐MOF (Figure 1f) thus possibly affecting the molecular exciton diffusivity within the ligand network through non‐radiative homo‐molecular energy transfer‐mediated hopping. Another evident difference is the final size and morphology of the synthesized MOF crystals. Scanning electron microcopy (SEM) images (Figure 1g) show micrometer‐size lamellar‐shaped crystals for In‐MOF and an octahedral morphology of nanocrystals centered at 70 nm for Zr‐MOFs and 390 nm for Hf‐MOFs (Figure S10). The samples are thermally stable up to 425°C for the In‐MOF and 475°C for the Zr‐ and Hf‐MOF.

### 2.2 Photoluminescence and Scintillation Properties

Considering that $\mathrm{LY}\propto \;\chi \phi_{\mathrm{pl}}$, where χ is the yield of conversion from ionized charges to emissive states and $\phi_{\mathrm{pl}}$ is the photoluminescence quantum efficiency, the influence of composition and the reciprocal spatial orientation among the ligands on the MOF emission properties were first investigated by photoluminescence experiments.

**FIGURE 2:**
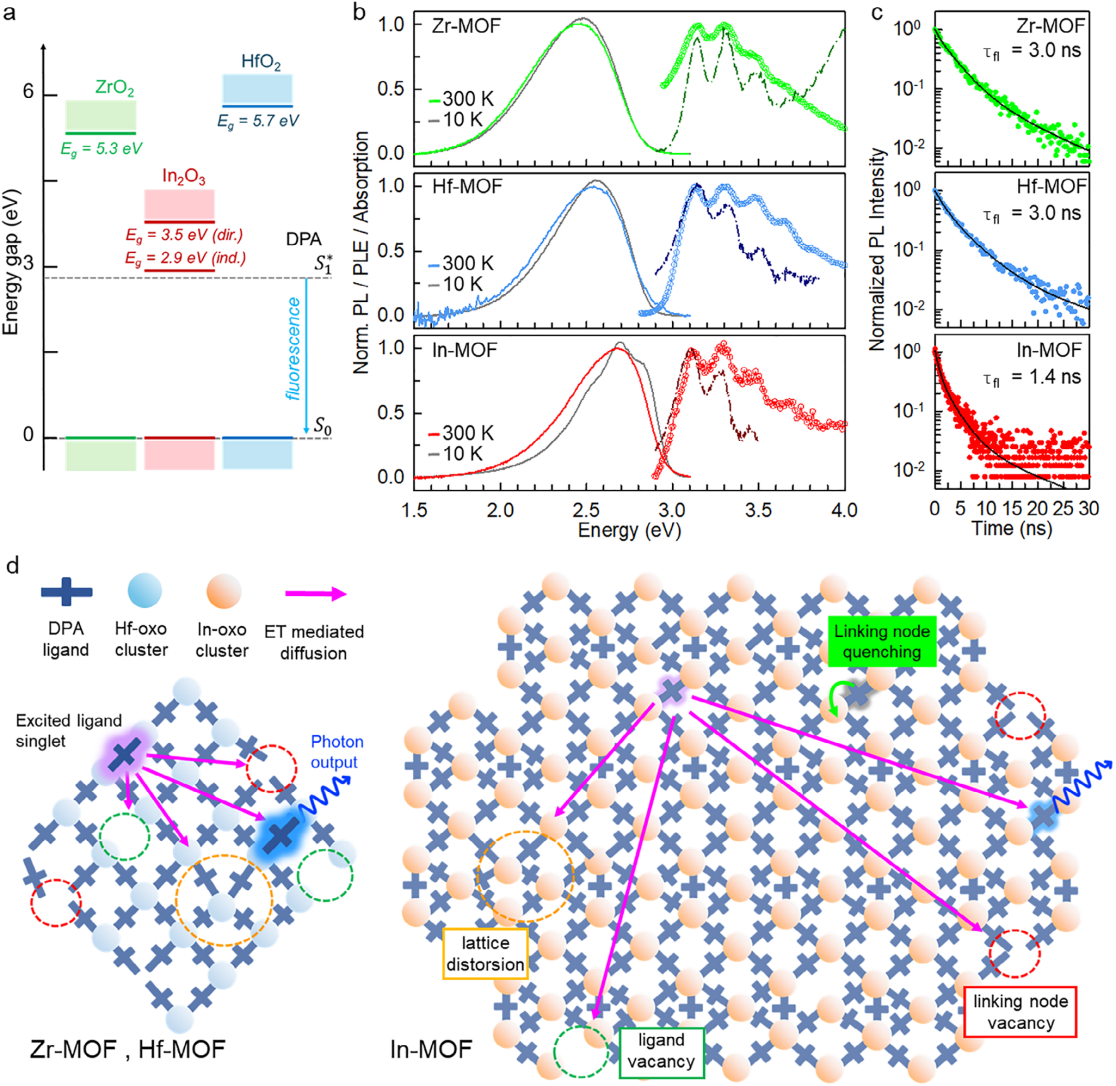
(a) Ground state electronic structures of the metal‐oxide cluster linking nodes and of the DPA ligand employed to synthetize scintillating MOFs. (b) Normalized optical absorption (dash‐dot line), photoluminescence (PL, solid line) and excitation PL (PLE, recorded at the PL maximum, dotted line) for the series of the MOF powders investigated. PL spectra recorded at 300 K and 77 K under excitation at 3.65 eV (340 nm). (c) Fluorescence intensity decay in time of MOFs, recorded under pulsed excitation at 3.65 eV at the PL maximum. Solid lines are the fit of data with a multi‐exponential decay function. (d) Sketch of the MOF crystalline structures and of the ligand singlet exciton diffusion and quenching process happening during its lifetime in Zr (or Hf)‐ MOF and in the larger In‐MOF. Dashed circles highlight possible defects in the MOF crystalline structure towards which the diffusing ligand exciton can move and where they recombine radiatively. These defects represent the intrinsic static disorder of electronic energies outlined by temperature dependent PL experiments. In addition, in the In‐MOF the presence of specific In‐oxo clusters’ electronic levels acting as quenchers of the emission of ligands is evidenced.

Figure 2a reports the energetic landscape of lower electronic levels for the building blocks of the MOF series, namely the metal nodes and the conjugated ligand DPA. Notably, both the Zr‐ and Hf‐oxo‐hydroxy clusters exhibit an energy gap wider than that from the singlet excited state $S_{1}^{*}$ to the singlet ground state *S*_0_ of the pure DPA ligand at 2.83 eV [61, 62, 63, 64], which generates the MOF luminescence. Conversely, the In‐based cluster has a narrower gap, specifically, in In_2_O_3_ nodes there is a direct and optically active energy gap of ≈3.50 eV. Moreover, a further indirect and optically dark gap is reported at 2.90 eV, which is still thermodynamically accessible at room temperature from the ligand $S_{1}^{*}\to S_{0}$ transition at 2.85 eV [65, 66]. Figure 2b shows the absorption, photoluminescence and excitation photoluminescence (PLE) spectra of the three MOFs as crystalline powders at room temperature (Methods). All the absorption spectra in the UV–Vis region show the typical vibronic structure of the isolated conjugated ligand (Figure S11), suggesting the absence of significant formation of undesired aggregated species. Under excitation at 3.65 eV (340 nm) all powders show a broad emission in the visible spectral range, ascribed to the recombination of DPA emissive singlets [15, 67]. The Zr‐ and Hf‐MOF emission spectra are smooth and peaked at 2.48 and 2.56 eV, respectively, while the In‐MOF shows an emission peaked at 2.70 eV with a pale similarity with the isolated ligand photoluminescence typically peaked at 2.87 eV (Figure S11). Thus, all the emission spectra show a red‐shift with respect to the isolated ligand emission spectrum. This is partially due both to reabsorption effects in the powder form, as observed for the crystalline powders of the pure ligand (Figure S12), and to the presence of a broad distribution of emitting states with energies slightly lower than the single molecule $S_{1}^{*}$ state which contributes to the overall emission [67].

The $\phi_{pl}$ values were measured using and integrating sphere, obtaining 0.42±0.04 and 0.41±0.04 for Zr‐ and Hf‐MOFs, respectively, and 0.19±0.03 for In‐MOF (Methods). The characteristic decay time of the fluorescence intensity at the emission maximum is τ_fl_ = 3.0±0.1 ns for Zr‐ and Hf‐MOFs and 1.4±0.1 ns for In‐MOF (Figure 2c). The recorded fluorescence decay times reflect a partial quenching of the MOF luminescence that lowers their $\phi_{\mathrm{pl}}$ with respect to that of the single ligand. Indeed, from the time resolved experiments we can estimate the $\phi_{pl}$ value as τ_fl_/τ_0_, where τ_0  _= 6.6±0.1 ns is the ligand emission lifetime as single molecule in absence of quenchers (Figure S11) [68]. We obtain a $\phi_{\mathrm{pl}}$ of 0.45±0.02 for Zr‐ and Hf‐MOFs and 0.21±0.02 for In‐MOF, respectively, in good agreement with those directly measured. The PLE spectra recorded at the maximum of the emission match the absorption ones (Figure 2b), thus demonstrating that the controlled framing of constituting chromophores avoids strong ground‐state intermolecular interactions, which would result in an optical behavior substantially different from that of the isolated molecules [69].

Upon cooling down the samples to 10 K, both Zr‐ and Hf‐based MOFs show a blue‐shift of the emission of 0.03  and 0.02 eV, respectively, as previously observed due to the narrowing of the emission and absorption bands at low temperature that reduces self‐absorption (Figure 2b) [67]. However, the emission is still clearly broad and smooth without any clear vibronic structure. These findings suggest that luminescent singlet excitons in Hf‐ and Zr‐based MOFs sense an intrinsic static disorder in the distribution of the electronic excited state energies, independent from the temperature, probably due to a broad distribution of local environments at the nanometric scale [15, 16, 39, 54]. Structural investigations are not sensitive enough to properly highlight the localized defects responsible for the static energetic disorder in crystals because of their low amount. However, as usual in high diffusivity crystalline systems, these defect‐related states are anyhow accessible by the diffusing molecular excitons, thus affecting the MOF global luminescence properties (Figure 2d, left). Conversely, the In‐MOF exhibits a radically different behavior. By lowering the temperature, we observe a slighter blue shift of 0.01 eV of the emission maximum and a clear vibronic structure appears in the photoluminescence spectrum (Figure 2b, bottom panel) matching the DPA ligand emission (Figure S11). From an electronic perspective, this result indicates that molecular excitons in this MOFs mainly sense a temperature‐dependent dynamic disorder in the distribution of the electronic excited state energies coupled to the phonons bath at room temperature [70], while the static disorder seems to be negligible. These findings suggest that In‐based nodes enable the growth of a material energetically more ordered with respect to the Zr‐ and Hf‐based systems. This is consistent with the fact that the effect of surface defects can be partially hindered in the larger In‐MOF crystals (Figure 2d, right) and there are no residual molecules of the modulator. Interestingly, the In‐MOF shows the lowest $\phi_{\mathrm{pl}}$ and the shorter emission lifetime, whilst the smaller Zr‐ and Hf‐MOF nanocrystals which still contain some modulator (12 mol% acetate and 5 mol% formate, respectively) and have a larger surface‐to‐volume ratio, two factors that are usually detrimental for emission, exhibit a better performance. These findings suggest that exciton diffusion in the In‐based MOF is faster, thus reaching more efficiently the available quenching centers.

**FIGURE 3:**
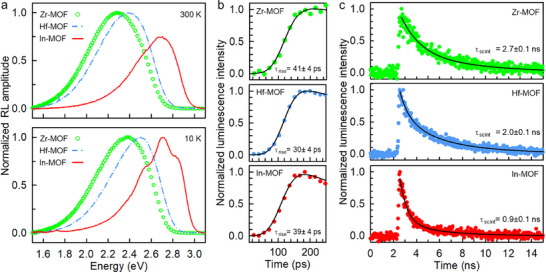
Normalized radioluminescence (RL) spectra of the crystalline powders of MOFs recorded under soft X‐ray excitation at 300 K (top panel) and 10 K (bottom panel). (b, c) Scintillation pulses intensity rise (b) and decay rise (c) under excitation with pulsed soft X‐rays at 14.5 keV, recorded at the luminescence emission maximum. Solid lines are the fit of the data with a multi‐exponential decay function convoluted to the X‐rays excitation pulse.

Radioluminescence and scintillation experiments under soft X‐rays enable to shed more light on the origin of the composition‐dependent luminescence features of MOFs. The MOFs emission is fully preserved after storage in open air for several months (Figure S12) [57]. No difference is observed in the radioluminescence spectra with respect to the photoluminescence, both at room temperature and 10 K (Figure 3a). Emission is completely stable up to 5 Gy of delivered dose (Figure S13) and no afterglow is detected for any of samples (Figure S14). At 300 K, the powders show a LY as high as ca. 4500±900, 1500±300 and 750±150 ph MeV^−1^ for Hf‐, Zr‐ and In‐MOFs, respectively (Methods). Remarkably, the Hf‐MOF shows a LY three times larger than that of Zr‐MOF. Since Hf has a higher atomic number than Zr (*Z*_Hf_ = 72 ~ 2*Z*_Zr_ = 80), the Hf‐MOF possesses an enhanced photoelectric interaction probability and, especially, an enhanced stopping power for the primary and secondary electrons which activate the scintillation [71]. The consequent enhanced localized generation of ionized charges in the neighborhood of the denser Hf‐oxo‐hydroxy clusters induces the observed LY increment by limiting the Onsager losses typical of low density scintillators [38, 57, 72]. This picture is supported by considering the kinetics of the scintillation pulses depicted in Figure 3b,c. The scintillation pulse intensity rise time is 30±4 ps in Hf‐MOF versus 41±4 ps in Zr‐MOF, thus demonstrating faster and more efficient generation of the ligands singlets in the Hf‐based system. Interestingly, also the pulse intensity decay kinetic is different: a characteristic decay time of 2.7±0.1 ns is observed for Zr‐MOF, basically unchanged with respect to photoluminescence, whilst a shorter 2.0±0.1 ns decay time is measured for the Hf‐MOF.

Considering that the two MOFs exhibit the same crystal structure with consequently similar exciton diffusivity (Section S3) and the same emission spectra and same $\phi_{\mathrm{pl}}$, the accelerations in the scintillation rise and decay time can be ascribed to the larger density of charges produced by X‐rays in the Hf‐MOF because of its higher *Z*. A larger density of charges increases the generation rate of ligand singlets, thus slightly accelerating the pulse rise time, but it simultaneously enhances the probability of luminescence quenching through singlet‐singlet annihilation (SSA) in the high diffusivity crystalline framework (Section S3). The SSA rate in rapid diffusion systems is proportional to the number of diffusing excited states [S*] and to their diffusivity *D*, *k*_SSA_∝ *D*[S*] (Section S3.1) [73]. The SSA rate can be estimated from experimental data as *k*_SSA_ = *k*_scint_ – *k*_fl_ = (*τ*_scint_)^−1^ – (*τ*_fl_)^−1^, thus obtaining a *k*_SSA_of 0.04 and 0.17 GHz for Zr‐ and Hf‐MOF, respectively. The obtained results on the LY indicate therefore that the presence of Hf ions boosts the probability to generate emissive states overcompensating the partial quenching of the emission by SSA, and thus resulting in a faster emission with higher yield with respect to the Zr‐MOF as recently observed in other systems [57].

The In‐MOF shows a radioluminescence strictly resembling its photoluminescence, and again at 10 K the emission spectrum matches the one of the isolated ligand. However, it shows a six‐fold lower LY with respect to the other MOFs. The poor scintillation efficiency cannot be ascribed solely to its lower $\phi_{\mathrm{pl}}$ value. Indeed, considering that *Z*_In_ ~ *Z*_Zr_, we expect a rate and efficiency for the emissive state generation similar to the Zr‐MOF. The scintillation rise time of 39±5 ps (Figure 3b, bottom) suggests that the presence slightly heavier In ions does not affect significantly the production and distribution of ionized charges as in the case of Hf. However, the scintillation intensity decay time is 0.9 ns, i.e., one third of the Zr‐MOF value. We partially ascribe this effect to the SSA mechanism. From data, the SSA rate in the In‐MOF can be estimated as large as *k_SSA_* = 0.40 GHz. Notably, this value is more than twice that in Hf‐MOF, thus suggesting again that a faster diffusion of singlet exciton in the In‐MOF that promotes both the photoluminescence quenching and the SSA rate. This picture is supported by the modeling of exciton diffusion in the different MOFs (Section S3). Since we consider singlet excitons, we can, in first approximation, predict the Förster homo‐molecular energy transfer that rules their motion within the ligand framework. We obtain a 190 GHz hopping rate within the ligands for the Zr‐ and Hf‐MOFs, which share the same crystalline structure, while a 112 GHz hopping rate is found for the In‐MOF. These values are in the same range, but, in general, they can underestimate the real hopping rate, which must include the Dexter energy transfer contribution that cannot be calculated a priori. We know from previous studies that the Dexter contribution to the singlet diffusivity in the Zr‐ and Hf‐MOFs is negligible [52], in agreement with the spatial arrangement of ligands that quite well separate their conjugated cores minimizing the short‐range exchange interaction that rules the Dexter mechanism [74, 75]. Conversely, the Dexter contribution is of paramount importance for the In‐MOF, where inter‐ligand distances are shorter than 1 nm making short‐range exchange interactions fully active. Indeed, by combining time resolved photoluminescence and scintillation data with the modeling of the homo‐molecular Förster energy transfer in the ligand frameworks, we obtain for the In‐MOF a $k_{Dx}^{\mathrm{DPA}-\mathrm{DPA}}$ as large as 4.59 THz (Section S3.1). This large value highlights the pivotal role of Dexter transfer in the In‐MOF where, despite the more compact structure, the Forster interaction is less effective than in the Zr‐ and Hf‐MOF due to an unfavorable reciprocal orientation of ligands (Section S3). Nevertheless, the observed luminescence quenching is still not large enough to justify the six‐time lower LY of the In‐MOF with respect to the other crystals. We ascribe this discrepancy to the occurrence of competing charge trapping processes in addition to non‐radiative recombination pathways [76, 77, 78], which can be investigated by temperature‐dependent experiments.

### 2.3 Temperature Dependent Luminescence Properties

**FIGURE 4:**
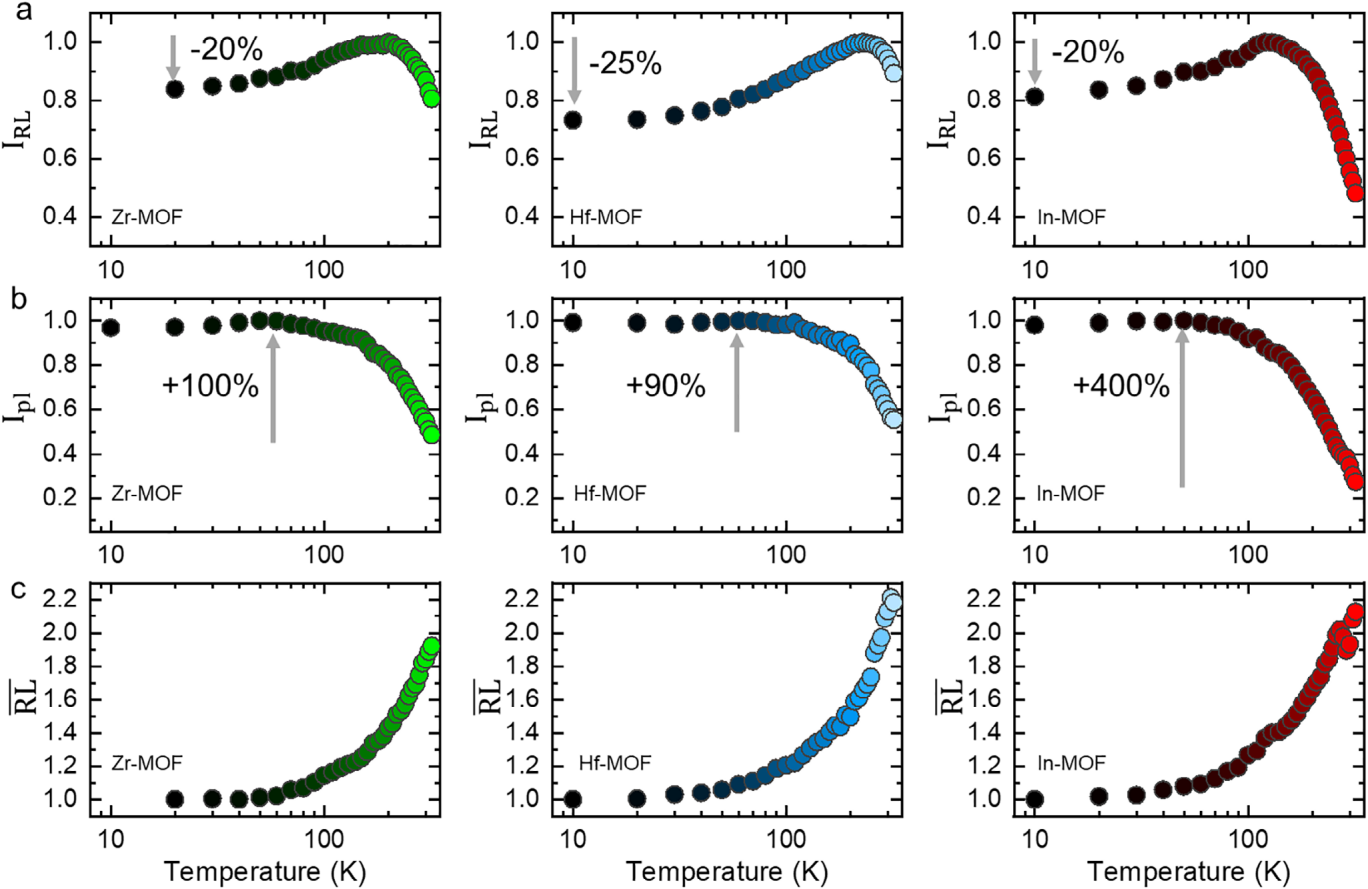
(a) Integrated radioluminescence intensity (*I*_RL_), (b) photoluminescence intensity (*I*_pl_) and (c) corrected radioluminescence intensity ($\bar{\mathrm{RL}}$ = *I*_RL_ /*I*_pl_) measured as a function of the temperature for Zr‐MOF, Hf‐MOF and In‐MOF powders under soft X‐rays (RL) or cw laser excitation at 261 nm (PL).

Figure 4 shows the integrated radioluminescence (*I*_RL_) and photoluminescence (*I*_pl_) intensity measured on the MOF powders by cooling the samples from 320 to 10 K (Figure S16). The *I*_RL_ has a clearly different behavior with respect to *I*_pl_. For all samples, *I*_RL_ has a non‐monotonic behavior, with an initial increment from 320 K until a temperature of ca. 200–100 K is reached. This is particularly evident in the In‐based system, where the *I*_RL_ value is almost doubled at 130 K. Below 100 K, all samples show the same decrease of 20–25% for the maximum I_RL_ value, reaching a plateau below 30 K. The initial rise of I_RL_ seems strictly correlated to the *I*_pl_ behavior. Indeed, the *I*_pl_ value increases significantly in all samples down to 80 K where the maximum emission intensity is reached. Specifically, at low temperature, the *I*_pl_ doubles in Hf‐ and Zr‐MOF and a huge +400% increment is observed for the In‐based system. This finding confirms a significant role of thermal quenching in the emission properties of all MOF, but to a significantly different extent in Hf‐ and Zr‐MOF versus the In‐MOF architecture.

**FIGURE 5:**
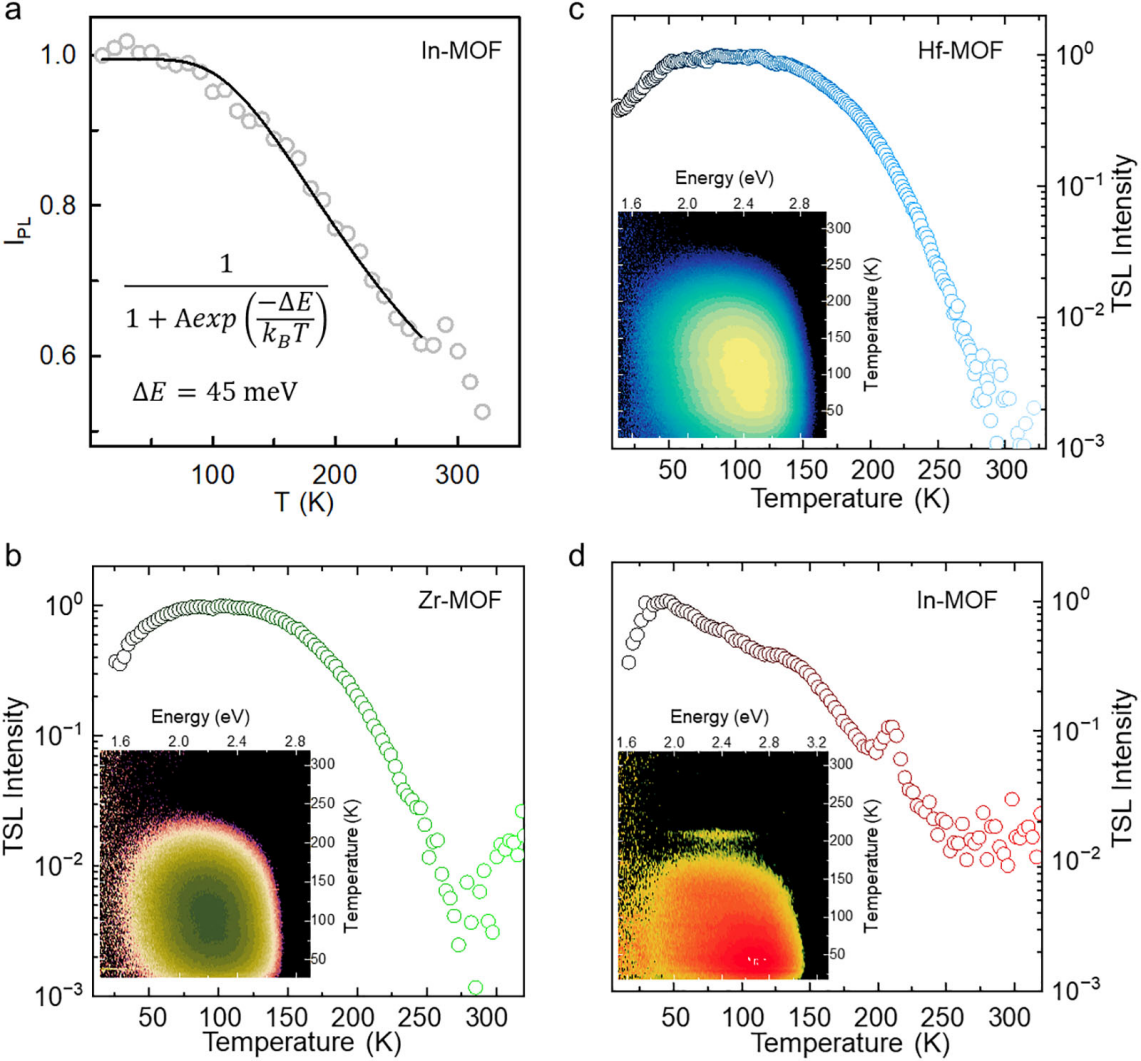
(a) Arrhenius plot of the photoluminescence quenching *I*_pl_ of the In‐MOF as a function of the temperature. (b–d) Glow curves for the (b) Zr‐MOF, (c) Hf‐MOF and (d) In‐MOF powders extracted by integrating the spectrally resolved thermally stimulated luminescence (TSL, insets) as a function of temperature the 1.8‐2.7 eV energy region after X‐ray irradiation at 10 K.

Considering the purely electronic origin of the mechanisms that rule the exciton diffusion (Förster and Dexter homo‐molecular energy transfer) in the MOF structures where ligands are framed in almost fixed positions, we do not expect that lowering the temperature can effectively block the singlet diffusion towards quenching sites. Therefore, at a first look the data in Figure 4 suggest that the emissive state energy at room temperature could be dissipated through non‐radiative intramolecular internal conversion [79], but the framing of highly emissive ligand significantly limit this pathway. However, for the In‐based system we must also consider the resonance of the ligand emissive transition $S_{1}^{*}\to S_{0}$ with the metal‐node dark energy gap at 2.90 eV. The distance between the ligand center and the nodes is about 1 nm (Figure S4). This length is compatible with a ligand‐to‐node non‐radiative energy transfer, that can be a good candidate as the responsible of the low $\phi_{\mathrm{pl}}$ and short emission lifetime of the In‐MOF. Notably, this transfer process is endothermic, with a *ΔE* ≈ 2.90 eV (In‐oxo‐hydroxy cluster) – 2.85 eV (ligand fluorescence) = 50 meV comparable to the thermal energy at room temperature. Therefore, at 10 K the transfer should be significantly limited according to the Boltzmann distribution, resulting in a remarkable enhancement of the photoluminescence intensity, as observed [80]. Figure 5a shows the Arrhenius plot for the quenching of the In‐MOF photoluminescence, corrected by the intramolecular vibrational quenching contribution observed in the Zr‐ and Hf‐based systems. The slope of the straight line that fits the data allowed us to estimate an activation energy of 45 meV for the non‐radiative energy transfer from the ligands to the In‐oxo‐hydroxy dark state. This result is in excellent agreement with the expected value, thus suggesting that this is the main quenching pathway for the In‐MOF photoluminescence at room temperature.

Lastly, by correlating the *I*_RL_ and the *I*_PL_ values we point out the effect of the temperature on the formation of the ligand emissive states during scintillation. Figure 4c depicts the evolution of the corrected radioluminescence intensity $\bar{\mathrm{RL}}$ = *I*_RL_ /*I*_pl_ values as a function of the temperature. In all MOFs, at 10 K we observe a similar drop of $\bar{\mathrm{RL}}$ to half of its initial value. This indicates that the generation of emissive states is positively affected by the high temperature, which helps to reduce the evident trapping of diffusing free carriers at cryogenic temperatures [81, 82]. To further investigate this aspect, wavelength‐resolved thermally stimulated luminescence (TSL) in the 10–320 K range were performed. The TSL contour plots of Zr‐MOF and Hf‐MOF (insets of Figure 5b,c) show a broad emission band peaked at around 2.2–2.4 eV, which matches their radioluminescence spectra (Figure 2), featuring a broad and continuous glow curve (Figure 5b,c) extending up to 220 K. This behavior is ascribed to the presence of a continuous distribution of defects acting as traps [76], thus confirming the energetic disorder for excited states pointed out by photoluminescence experiments. Despite the relative intensity of this traps‐related emission is less than 3% with respect to the total emission in all the investigated MOFs (Figure S17 and ref. [55]), its presence enables further analysis. By the initial rise method (Methods, Figure S18) and partial cleaning procedure, the energy depth of this trap distribution was extracted, which turned out to span from around 50 to 220 meV. On the other hand, the In‐MOF TSL results in a more structured glow curve (Figure 5d), evidencing a more defined trap state distribution in good agreement with the observed better energetic order for this system previously discussed.

To summarize, the obtained results suggest that the luminescence properties of Zr‐MOF and Hf‐MOF nanocrystals are set by a broad distribution of emissive defects. In the nanocrystalline Zr‐ and Hf‐MOFs, characterized by a higher surface‐to‐volume ratio, surface states and dangling bonds are fully accessible by fast diffusing singlets, and behave like low energy emitters/quenchers for excitons and charges [67, 81, 83]. This leads to a temperature‐independent broad distribution of the local environments sensed by molecular excitons (Figure 2d, left) which is superimposed to the intrinsic thermal broadening of electronic energies and dominates the luminescence properties. Conversely, this effect is mitigated in the In‐MOF due to the larger size of crystals, which limits the role of surfaces, thus showing luminescence properties mostly determined by bulk characteristics, such as the presence of the metal‐node dark state almost resonant with the ligand emission, which acts as the main quenching species that sets the system luminescence properties. It is worth noting that in systems with high diffusivity, such as the In‐MOF, reducing the size can introduce additional energy dissipation channels. Indeed, in smaller In‐MOFs, we should expect a more efficient quenching of the luminescence by the more easily accessible surface defects. In this case, a proper surface treatment will be essential to obtain highly emissive MOFs [67].

## 3 Conclusions

We investigated the luminescence properties of a series of MOFs based on high‐Z metal‐oxo‐hydroxy cluster and fluorescent conjugated ligands, which were designed as fast scintillators. Three MOFs were selected in order to i) point out the effect of the Z‐number of the linking nodes whilst maintaining the same crystal structure, and ii) the effect of a different crystalline structure by keeping almost unchanged the MOF composition. By increasing the atomic number in MOFs from Zr‐ to Hf‐oxo‐hydroxy clusters, we observed that the photoluminescence yield remained unchanged, as set by thermal and diffusion‐mediated singlet exciton quenching, while a significant increase in scintillation speed and efficiency, about +300%, is recorded. This is a remarkable result, which highlights the influence of higher Z elements to enhance the material stopping power, the charge‐to‐emissive state conversion and the scintillation kinetics, despite the average density of the MOF remains substantially unchanged.

The use of metal nodes with comparable atomic number *Z*, such as Zr and In, but different coordination geometries, enabled the realization of distinct arrangements of the DPA emitters. In the investigated case, the In‐oxo‐hydroxy cluster generates a closer arrangement of ligands favored by the triply interpenetrated architecture, which boosts the fluorescent molecular excitons diffusivity, by activating a more efficient bimolecular quenching of luminescence. This makes the In‐MOF a sub‐nanosecond fast scintillator. However, the scintillation and photoluminescence yields of the In‐MOF are severely limited by thermally activated energy transfer towards the dark state of the In‐clusters, which dominates excited ligand radiative recombination at room temperature. This finding highlights the important role of the electronic structure of the linking nodes, which should be considered as important as the accessibility of surface defects in high diffusivity crystalline systems, such as MOFs.

These results clearly highlight the extreme versatility of the MOF platform to realize optically active luminescent and scintillating materials. Importantly, this study clearly points out for the first time the crucial role played by the fine engineering of the metal‐node electronic properties, in addition to that of the ligands, to realize highly luminous and fast emitters, an aspect rarely considered. Therefore, the control and tuning of linking‐node optical and electronic properties and their interplay with ligands should be effectively considered to design the next generation of advanced MOF‐based scintillators with tailored timing and spectral properties to meet specific technological demands.

## 4 Methods

### 4.1 MOFs Synthesis

#### 4.1.1 Zr‐MOF Synthesis

^51^ Zirconium chloride (ZrCl_4_, 116.5 mg; 0.50 mmol) and 4,4′‐(anthracene‐9,10‐diyl) dibenzoic acid (DPA) (209 mg; 0.50 mmol) were dispersed in a mixture of dry DMF (50 mL) and deionized water (50 µL) in a 100 mL glass vial and 1.43 mL (25 mmol) of acetic acid was added. The mixture was sonicated for 1 min at room temperature and then heated to 120°C for 22 h. The vial was cooled down to room temperature and the slightly yellowish solid was separated by filtration on a 0.2 µm PTFE membrane and washed with DMF (3×100 mL) and then CHCl_3_ (3×100 mL). The sample was activated at 120°C overnight under high vacuum. Yield: ≈50%.

#### 4.1.2 Hf‐MOF Synthesis

^53^ 4,4′‐(anthracene‐9,10‐diyl) dibenzoic acid (DPA)(209.0 mg; 0.50 mmol) and HfCl_4_ (160.0 mg; 0.50 mmol) were added to a 100 mL Pyrex bottle with a cleavable Teflon‐lined cap. Dry DMF (50 mL) and 400 µL of formic acid were added and the bottle was closed and sonicated for 1 min to obtain a well‐dispersed mixture. The mixture was heated to 120°C for 22 h in a preheated oven. The glass bottle was then removed from the oven and cooled to RT. The yellowish solid was collected by filtration on a 0.2 µm PTFE membrane, washed with DMF (3×100 mL) and then CHCl_3_ (3×100 mL). The powder was recovered and dried at 120°C under high vacuum before further analysis. Yield: 178 mg (58%).

#### 4.1.3 In‐MOFs Synthesis

The ligand 4,4′‐(anthracene‐9,10‐diyl) dibenzoic acid (DPA) was synthesized following the literature procedures [84]. A mixture of DPA (20 mg, 0.048 mmol), indium chloride (InCl_3_) (40 mg, 0.18 mmol), dimethyl acetamide (DMAC) (2 mL), dioxane (0.5 mL), water(0.25 mL) and concentrated nitric acid (10 µL) was stirred in a 23 mL vial for 10 min. The reaction mixture was heated under autogenous pressure to 120°C for 3 d and then cooled to RT by 24 h period, the desired product appeared in ≈42% yield. The resulting solids were collected followed by sequential washing with fresh DMF three times. Chemical formula of the solid is assigned as [In_3_(C_28_H_16_O_4_)_3_O(H_2_O)_3_Cl]_n_·4DMAC from SC‐XRD, TGA and elemental analysis. Elemental analysis for [In_3_(C_28_H_16_O_4_)_3_O(H_2_O)_3_Cl]_n_·4DMAC: calcd. (%): C 58.66, H 4.43, N 2.74; found (%):C 58.79, H 4.10, N 2.43.

### 4.2 Materials Characterization

#### 4.2.1 Infrared Spectroscopy

Fourier transform infrared (FTIR) spectroscopy. FTIR spectra measurements were performed with a Jasco FT/IR 4100 equipped with an ATR PRO450‐S module. The spectra were accumulated 128 times between 600 and 4000 cm^−1^, with a resolution of 2.0 cm^−1^.

#### 4.2.2 Thermogravimetric Analysis (TGA)

TGA analyses were performed using a Mettler Toledo Star System 1 equipped with a gas controller GC10. TGA analysis performed under an oxidative atmosphere (dry air, flow rate = 50 mL min^−1^) from 30°C to 1000°C highlighted the thermal stability of the samples and it allowed the evaluation of the residue after degradation of the organic component.

#### 4.2.3 ^1^H Solution NMR Spectroscopy

^1^H‐NMR spectra were recorded on a AVANCE NEO Bruker instrument (400 MHz). MOF samples were digested with deuterated trifluoroacetic acid (TFA_d, 0.1 mL), and the solution was diluted with deuterated dimethyl sulfoxide (DMSO_d6, 0.8 mL).

#### 4.2.4 Scanning Electron Microscope (SEM)

SEM images were collected using a Zeiss Gemini 500 microscope, operating at 5 KV. The sample was deposited on a conductive tape and sputtered with gold (≈10 nm) before the analysis.

#### 4.2.5 Powder X‐Ray Diffraction, Pawley and Rietveld Refinement

PXRD measurements of Zr‐MOF and Hf‐MOF were collected with a Rigaku Smartlab SE powder diffractometer over a range of 2θ of from 3.5 to 80.0° with a step size of 0.02° and a scan speed of 1.0° min^−1^ and 0.3° min^−1^, respectively, using Cu‐Kα radiation, at 40 kV and 30 mA. The Zr‐MOF and Hf‐MOF models for the Rietveld refinements were generated from the previously published Zr‐DPA and Hf‐DPA structures [52, 85]. Rietveld structural refinements of the X‐ray data were performed using the TOPAS‐Academic64 V6 software package [86]. For the Rietveld refinement, the structure was modelled as a disordered system with a space group *Fm*‐3*m*. The background was fitted and refined using a Chebyshev polynomial with 10 coefficients in the 2*θ* range from 3.5° to 80°. The “Simple_Axial_Model” was used to account for the asymmetry in the peaks, especially at low 2*θ* values. The peaks were fitted using a PearsonVII “ PVII ” function. Pawley refinement of the powder X‐ray data of In‐MOF was performed using the TOPAS‐Academic64 V6 software package, starting from the In‐MOF crystal structure determined from single‐crystal analysis [86]. Pawley refinement was performed between 4 and 35° 2 theta. The peaks were fitted using a PearsonVII “ PVII ” function.

#### 4.2.6 Photoluminescence Studies

Optical absorption spectra of MOF powders have been measured with a Perkin Lamda 900 spectrophotometer using an integrating sphere. Steady state PL spectra were excited using a UV laser at 261 nm (4.75 eV) the emitted light was collected using the same detection system as for radioluminescence measurements both at room and cryogenic temperature. The MOF powders photoluminescence quantum yield $\phi_{\mathrm{pl}}$ has been measured using an integrating sphere Hamamatsu C9920‐03G absolute photoluminescence quantum yield spectrometer with excitation at 360 nm (3.44 eV). Excitation photoluminescence (PLE) spectra have been recorded using a Cary 50 spectrophotometer in reflection geometry. Time resolved photoluminescence experiments have been performed using a 340 laser diode (3.65 eV, EP‐LED 340 Edinburgh Instruments) coupled to FLS980 Edinburgh setup in time‐correlated single photon counting (TCSPC) acquisition mode. The overall time resolution of the setup was 300 ps.

#### 4.2.7 Radioluminescence Studies

Radioluminescence measurements were carried out at room temperature using a homemade apparatus featuring, as a detection system, a liquid nitrogen‐cooled, back‐illuminated, and UV‐enhanced charge coupled device (CCD) Jobin‐Yvon Symphony II, combined with a monochromator Jobin‐Yvon Triax 180 equipped with a 100 lines/mm grating. All spectra are corrected for the spectral response of the detection system. RL excitation was obtained by unfiltered X‐ray irradiation through a Be window, using a Philips 2274 X‐ray tube with tungsten target operated at 20 kV. At this operating voltage, a continuous X‐ray spectrum is produced by a Bremsstrahlung mechanism superimposed to the L and M transition lines of tungsten, due to the impact of electrons generated through thermionic effect and accelerated onto a tungsten target. Cryogenic radioluminescence measurements are performed in the 10−320 K interval by lowering the temperature with a closed‐cycle He cryostat and controlling the temperature with a Lakeshore 330 temperature controller.

#### 4.2.8 Relative Light Yield Measurements

The relative light yield is obtained by direct comparison of the radioluminescence response of MOFs powders uniformly compacted in a cylindrical volume and commercial Bi_4_Ge_3_O_12_ bulk single crystal in powder form measured in the same experimental and geometrical conditions used as reference scintillator with a known light yield of 10 000 ph MeV^−1^. The thickness of the powder samples has been evaluated by the direct measurement of their mass and density, with an uncertainty of ± 2 µm, and is largely greater than the penetration depth of X‐rays, considering the X‐ray linear attenuation coefficient of around 60–70 cm^−1^ at the mean X‐ray energy of 7.2 keV [87]. This approach leads to an experimental uncertainty of 20% on the LY values.

#### 4.2.9 X‐Rays Scintillation Experiments

Time‐resolved scintillation spectra were collected by means of a Jobin Yvon Horiba MicroHR spectrometer coupled to a PicoHarp 300 multichannel scaler and a PMA Hybrid photomultiplier (Picoquant PMA‐07) as photodetector working in TCSPC mode (time resolution 4 ps). X‐ray pulses are generated using a 405 nm ps‐pulsed laser (Edinburgh Instruments, EPL‐ 405) to activate the photocathode of the N5084 Hamamatsu X‐ray tube with an applied voltage of 40 kV. Scintillation pulse simulations and fitting have been performed considering the deconvolution of the instrument response function (IRF, pulse width half maximum 140 ps) with rise and decay times.

#### 4.2.10 Thermally Stimulated Luminescence Measurements

Wavelength‐resolved TSL measurements at cryogenic temperatures are carried out by using the same detection system as for RL measurements. Cryogenic TSL measurements are performed in the 10−320 K interval, with a heating rate of 0.1 K s^−1^ after X‐ray irradiation at 10 K. The shape of the TSL signal versus T (the so‐called glow curve) has been corrected for the variation of the RL emission intensity versus T, to decouple the trap contribution to the emission from the other mechanisms involved in the scintillation process. The initial rise method is used for the evaluation of trap depth Δ*E*_T_. When the initial rising portion of the TSL glow curve is displayed against T^−1^, an exponential behavior is expected due to the Arrhenius‐like dependence of the TSL signal versus temperature at the beginning of trap emptying. The thermal energy of the trap level can, thus, be evaluated by the following expression: *I* (*T*)/*I*_0_ = 1/ [1+*A*exp(‐Δ*E*_T_/*k*_B_ *T*)] [88], where *I*(*T*) is the TSL intensity, Δ*E*_T_ the thermal energy, and k_B_ the Boltzmann constant. The above equation approximates the more complex relation between TSL intensity, temperature and trap parameters, and it is valid in the initial portion of the glow peak (up to ≈10% of the maximum intensity). Here the initial rise method is applied after partial cleaning procedures at three different temperatures, which comprised preheating up to *T* = 60 K, *T* = 100 K and *T* = 140 K, following the X‐ray irradiation at 10 K, after which the sample is cooled again before acquiring the TSL measurement.

## Conflicts of Interest

The authors declare no conflicts of interest.

## Supporting information




**Supporting File**: advs75224‐sup‐0001‐SuppMat.docx.

## Data Availability

The data that support the findings of this study are available from the corresponding author upon reasonable request.
