# Supplementary material for: Structural and Compositional Effects on the Scintillation Properties of Fast Emitting Metal‐Organic Frameworks
Source: Adv Sci (Weinh). 2026 Apr 16:e75224. Online ahead of print. doi: 10.1002/advs.75224 (PMC13334969; doi:10.1002/advs.75224)
Supplement: Supplementary file 1 — Supporting File: advs75224‐sup‐0001‐SuppMat.docx. [file ADVS-9999-e75224-s001.docx]

Supporting Information

Structural and compositional effects on the scintillation properties of fast emitting metal organic frameworks.

F. Cova^1^, J. Perego^1^, B. Joarder^2^, N. Yanai^3^, A. Vedda^1^, S. Bracco^1^, A. Comotti^1^, A. Monguzzi^1, 4^ & I. Villa,^1,4^*

F. Cova, I. Villa., J. Perego., A.Vedda, A. Comotti, A. Monguzzi

^1^ Department of Materials Science, University of Milano - Bicocca, Via Cozzi 55, I-20125, Milano, Italy

^2^B. Joarder,

Advanced Membranes & Porous Materials Platform (AMPMP), Division of Physical Sciences ‎and Engineering, King Abdullah University of Science and Technology, Thuwal, 23955-6900, Saudi ‎Arabia.‎

^3^N. Yanai

Department of Chemistry, Graduate School of Science, The University of Tokyo, 7-3-1 Hongo, Bunkyo-ku, Tokyo, 113-0033, Japan

^4^ NANOMIB BioNanoMedicine Center (https://nanomedicine.unimib.it/)

**Index**

**1. MOFs INVESTIGATION** page 2

1.1 Powder X-ray diffraction (PXRD) page 2

1.2 Thermogravimetric analysis (TGA) page 5

1.3 Infrared spectroscopy page 6

1.4. ^1^H solution NMR page 7

1. **Time resolved luminescence data Analysis** page 10
2. **EXCITON DIFFUSION MODELING IN MOFs** page 11
3. **additional LUMINESCENCE DATA** page 14
4. **SUPPLEMENTARY REFERENCES** page 21

**1. MOFs INVESTIGATION**

**1.1 Powder X-ray diffraction (PXRD)**


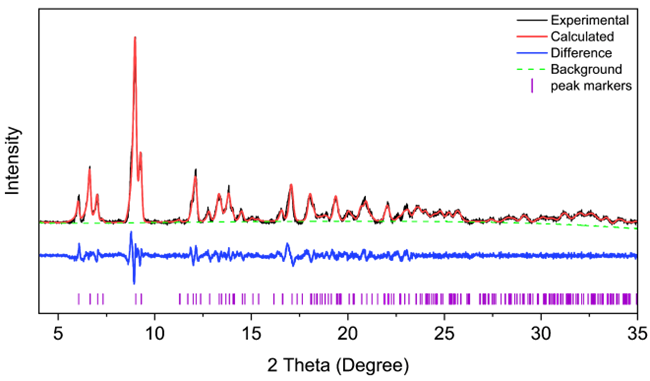


**Figure S1**. Experimental PXRD pattern and Pawley refinement plot for In-MOF.

**Table S1**. Crystal data and structure parameters for In-MOF calculated from PXRD data by Pawley refinement.

| Identification code | **In-MOF** |
| --- | --- |
| Method | P-XRD |
| Temperature/K | RT |
| Crystal system | Orthorhombic |
| Space group | *Cmcm* |
| a/Å | 25.068(3) |
| b/Å | 15.632(2) |
| c/Å | 29.121(3) |
| Volume/Å^3^ | 11411(2) |
| Radiation | CuKα (λ_avg_ =1.54184 Å) |
| 2Θ range for data collection/° | 4 to 35 |
| R indexes | Rp = 6.89%  Rwp = 9.23% |


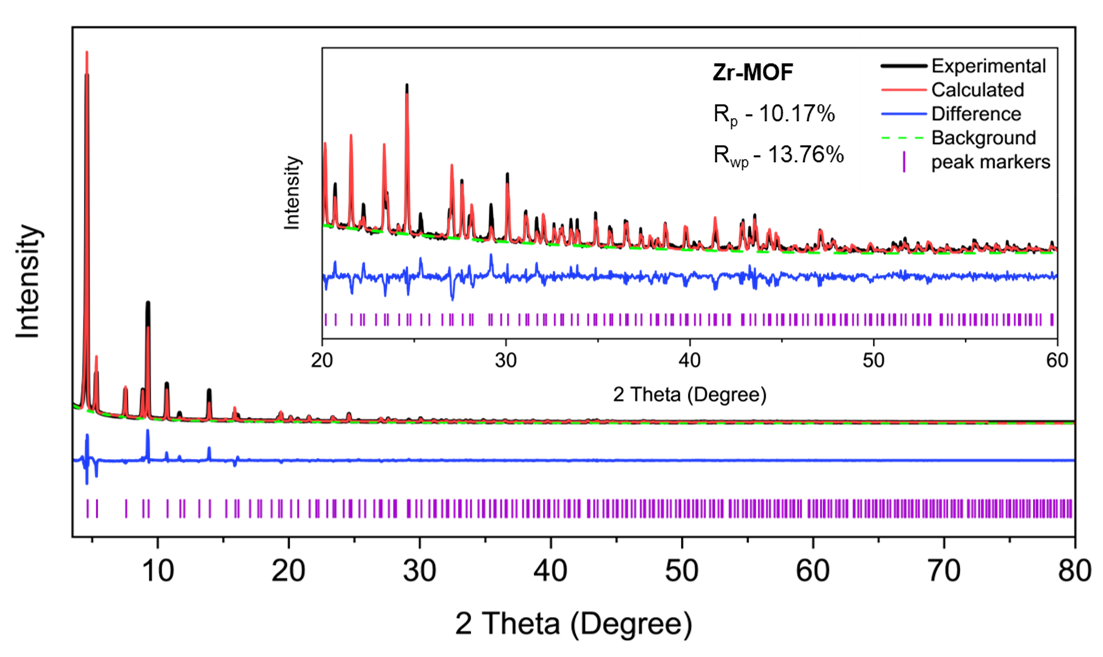


**Figure S2**. Experimental PXRD pattern and Rietveld refinement plot for Zr-MOF.


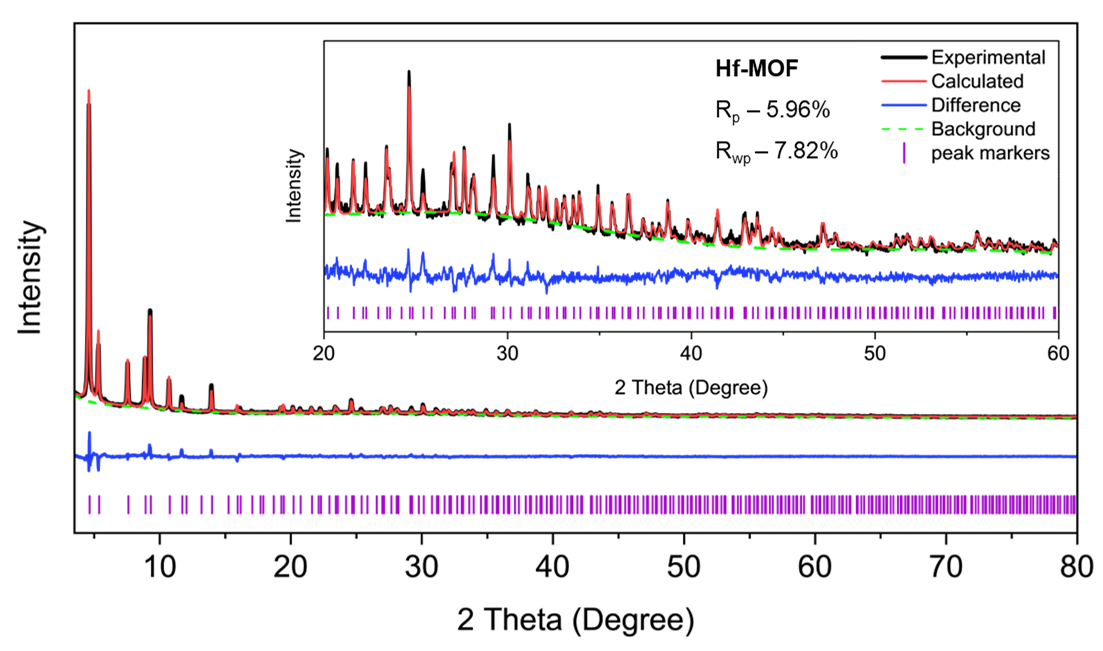


**Figure S3.** Experimental PXRD pattern and Rietveld refinement plot for Hf-MOF.


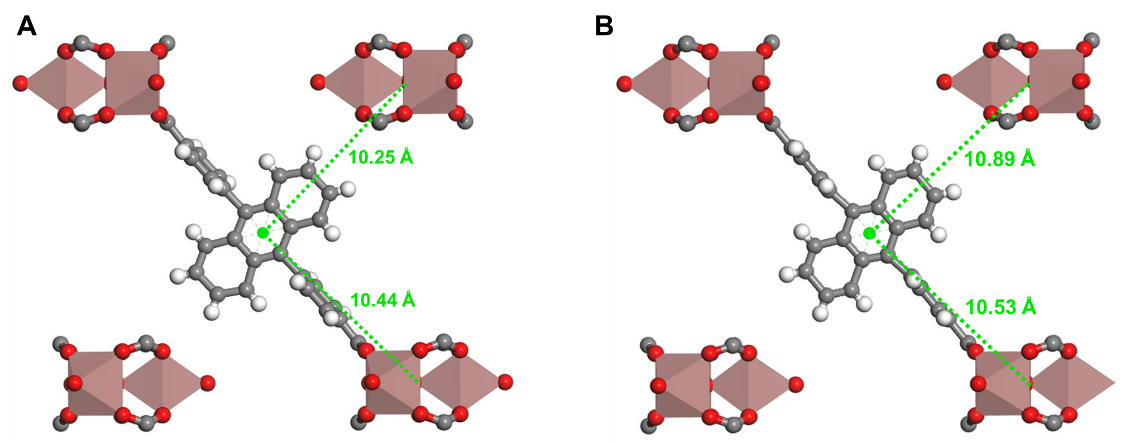


**Figure S4**. A)-B) Distances between the centroid of the anthracene molecules and the nearest-neighbour clusters in the In-MOF. The two independent DPA molecules in the crystal structure are reported in panels A and B, respectively.

**Table S2.** Crystal data and structure refinement for Zr-MOF and Hf-MOF resolved from P-XRD data by Rietveld refinement.

| Identification code | **Zr-MOF** | **Hf-MOF** |
| --- | --- | --- |
| Method | P-XRD | P-XRD |
| Empirical formula | C3.5 H2.083 O0.667 Zr0.125 | C3.5 H2.083 O0.667 Hf0.125 |
| Formula weight | 66.213 | 77.121 |
| Temperature/K | 298 K | 298 K |
| Crystal system | cubic | cubic |
| Space group | *Fm*-3*m* | *Fm*-3*m* |
| a=b=c/Å | 32.889(1) | 32.845(1) |
| Volume/Å^3^ | 35577(4) | 35440(4) |
| Z | 192 | 192 |
| Radiation | CuKα (λ_avg_ =1.54184 Å) | CuKα (λ_avg_ =1.54184 Å) |
| 2Θ range for data collection/° | 3.5 to 80 | 3.5 to 80 |
| R indexes | Rp = 10.17%  Rwp = 13.76% | Rp = 5.96%  Rwp = 7.82% |

**1.2 Thermogravimetric analysis (TGA)**


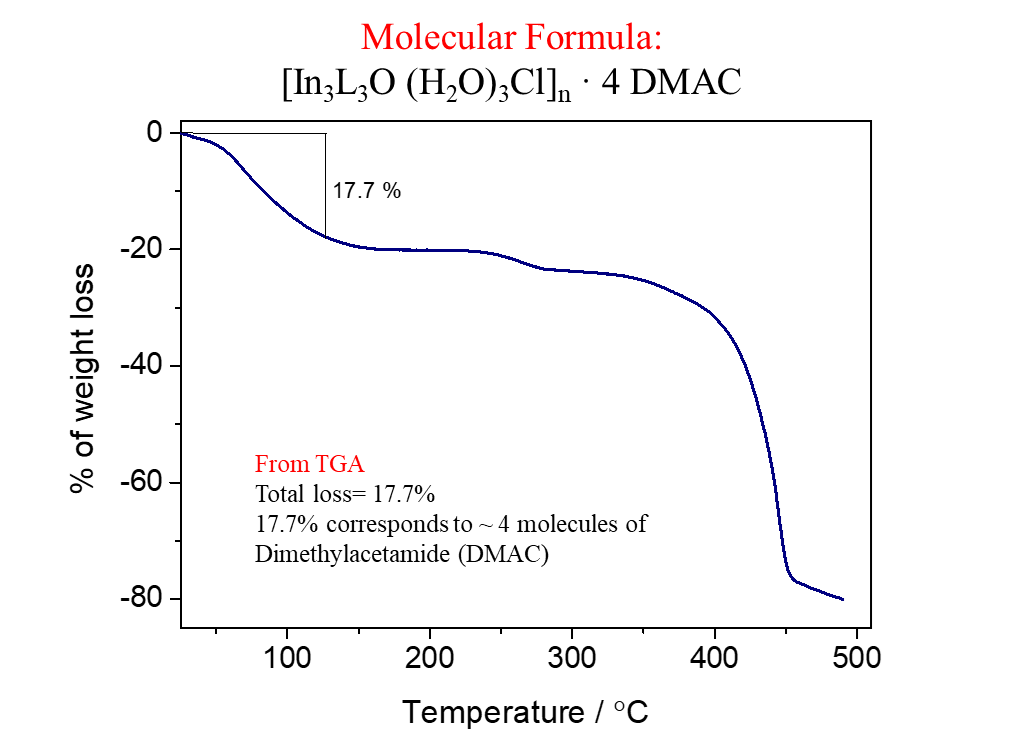


**Figure S5**. TGA analysis of In-MOF from 30°C to 500°C performed in air with a ramp rate of 10°C/min. In-MOF shows thermal stability up to 425 °C.


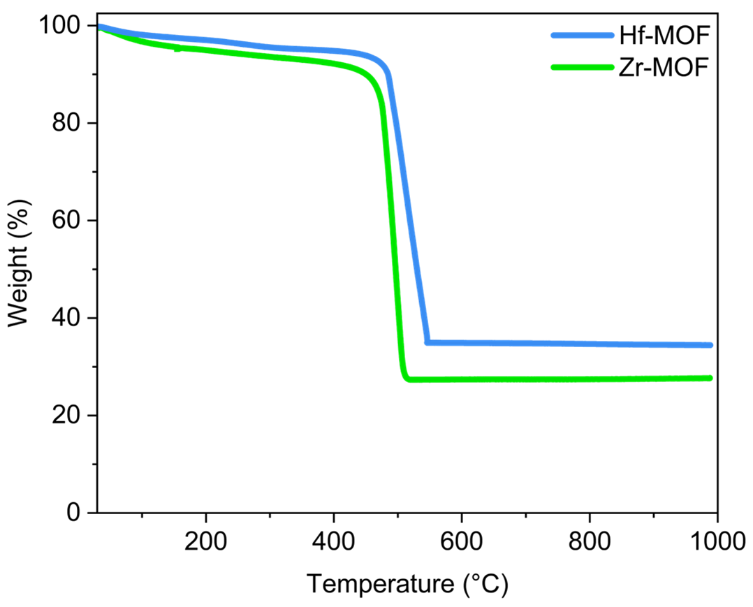


**Figure S6**. TGA analysis of Zr-MOF and Hf-MOF from 30°C to 1000°C performed in air with a ramp rate of 10°C/min. Zr-MOF shows thermal stability up to 450 °C. The experimental residue (25.6 % residual mass) is in good agreement with the theoretical value (23.3 %) calculated for the formation of ZrO_2_ after thermal treatment. Hf-MOF shows thermal stability up to 475 °C. The experimental residue (34.3 % residual mass) is in excellent agreement with the theoretical value (34.1 %) calculated for the formation of HfO_2_ after thermal treatment.

**1.3 Infrared spectroscopy**


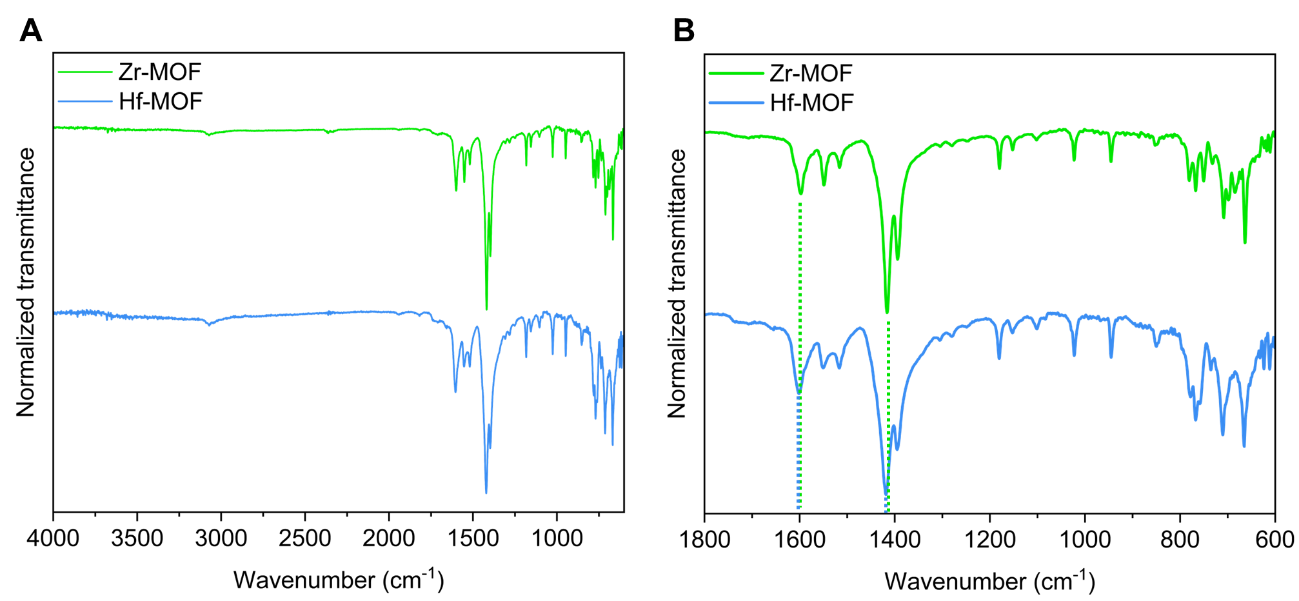


**Figure S7**. Comparison between the FT-IR spectra of Zr-MOF (green) and Hf-MOF (light-blue) between (A) 600 and 4000 cm^-1^ and (B) 600 and 1800 cm^-1^. The intense doublet at 1597 and 1415 cm^-1^ for Zr-MOF, and 1601 and 1418 cm^-1^ for Hf-MOF are associated with the in- and out-of-phase stretching modes of the carboxylate group coordinated with the oxo-hydroxy cluster.

**1.4. ^1^H solution NMR**


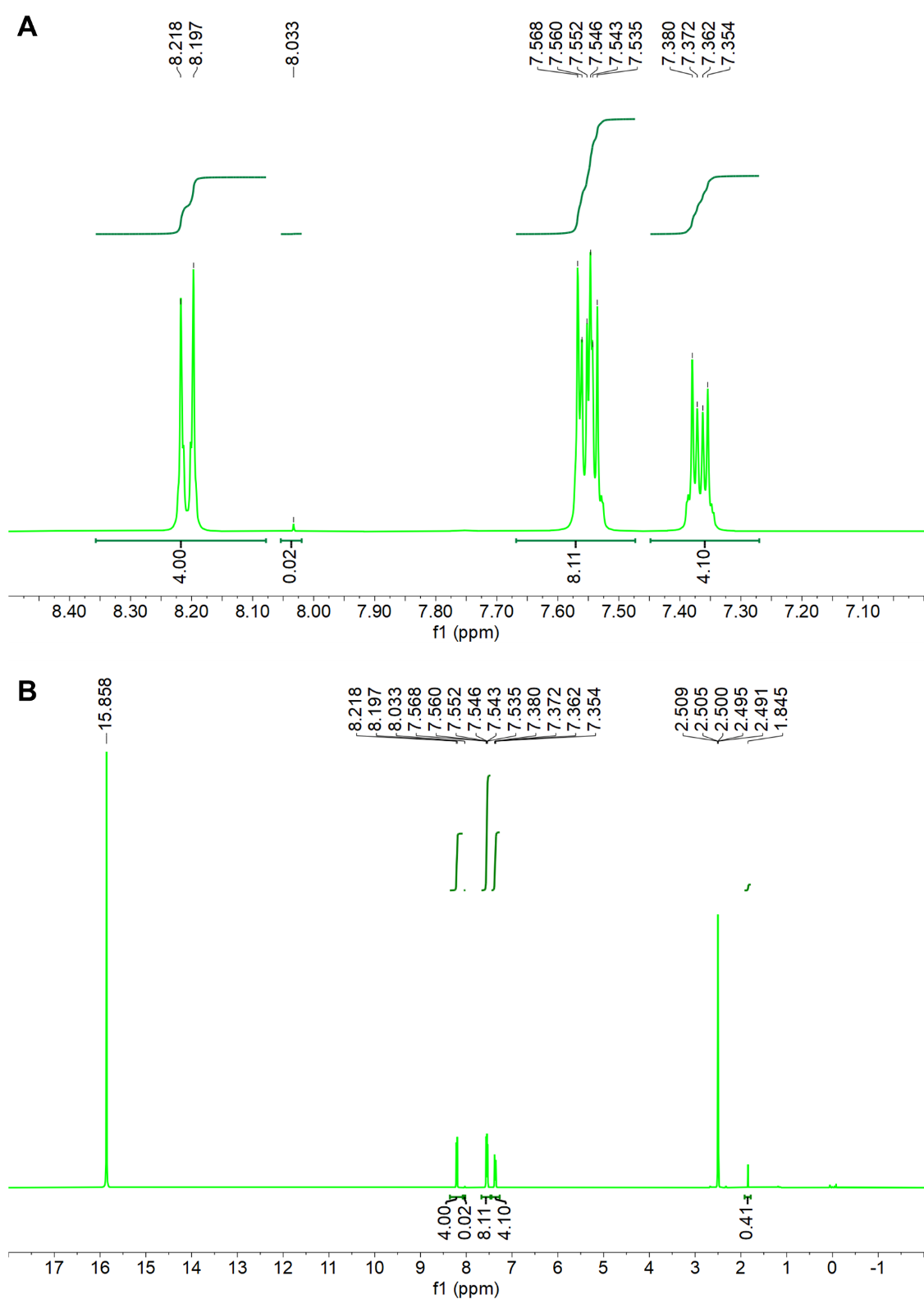


**Figure S8.** ^1^ H solution NMR for Zr-MOF after digestion under acidic conditions. The methyl group of the residual modulator (acetic acid) resonates at 1.845 ppm. The 12 % molar percentage of acetic acid included in Zr-MOF was evaluated from the integration of the signals of DPA and acetic acid.


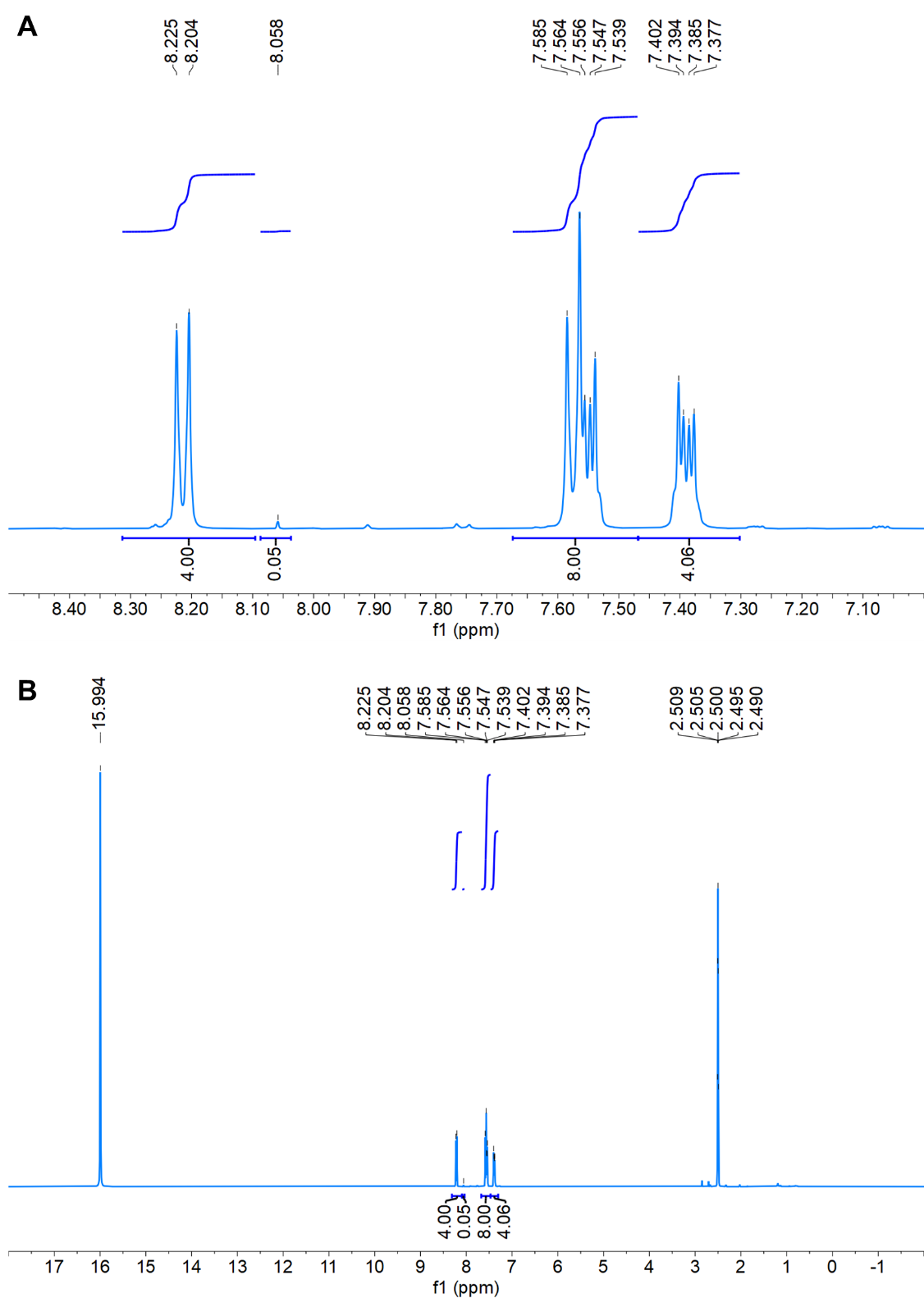


**Figure S9.** ^1^H solution NMR for Hf-MOF after digestion under acidic conditions. The methine group of the residual modulator (formic acid) resonates at 8.058 ppm. The 5 % molar percentage of formic acid included in Hf-MOF was evaluated from the integration of the signals of DPA and formic acid.

**1.5 Particle size distributions**


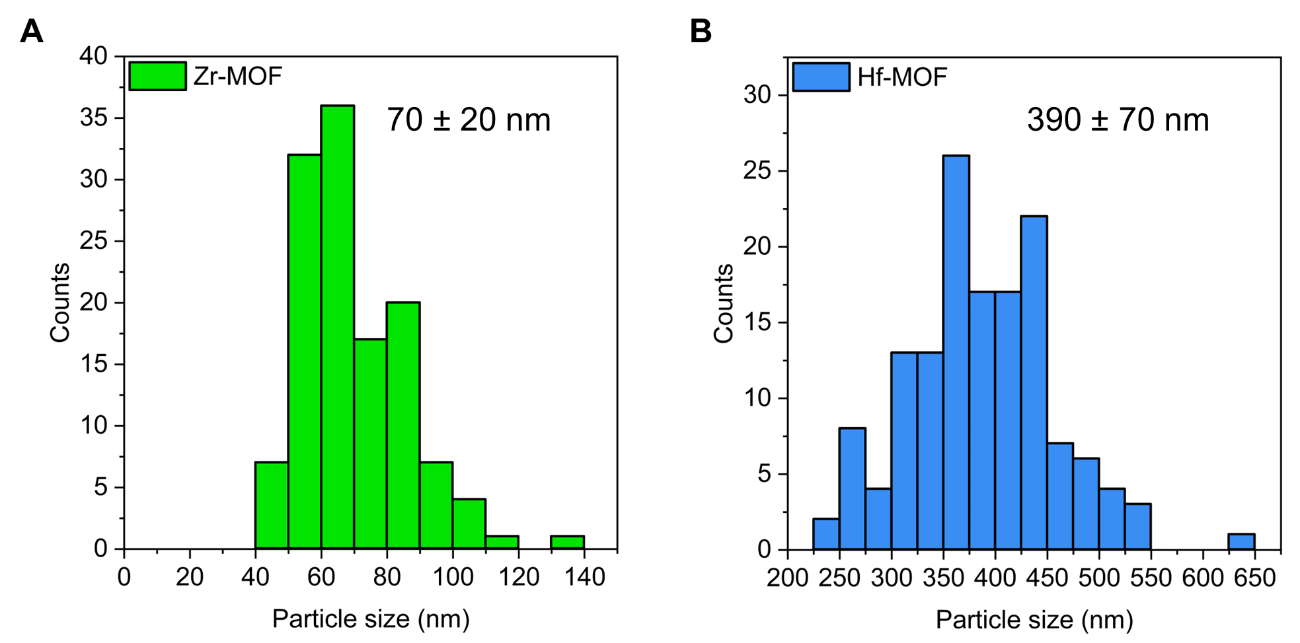


**Figure S10.** Particle size distribution calculated from SEM images for Zr-MOF (green) and Hf-MOF (light-blue).

1. **Time resolved luminescence data Analysis.**

The time resolved photoluminescence spectra and scintillation data discussed in the main text show in general a complex behavior. The signal decay has been reproduced with an analytical multi-exponential function

$I_{PL}\left( t \right) \propto\sum_{i} A_{i}e^{-\left( \frac{t}{\tau_{i}} \right)}$. Eq. S1

The parameters used for the fitting procedure are reported in Table 1. The characteristic emission lifetime $\tau$ has been calculated as the time at which the $I_{PL}$ is reduced to a 1/*e* of its initial value.

**Table S3.** Fit parameters employed to analyze the time resolved photoluminescence recorded on co-assembled MOFs dispersions in THF and scintillation experiments on co-assembled MOFs nanocomposites.

| @ PL max. | *τ_1_* (ns) | A_1_ | *τ_2_* (ns) | A_2_ | *τ* _3_ (ns) | A_3_ | *τ* _1/e_ (ns) |
| --- | --- | --- | --- | --- | --- | --- | --- |
| MOF |  |  |  |  |  |  |  |
| Zr-MOF | 1.6 | 0.40 | 4.7 | 0.55 | 19.6 | 0.05 | 3.0 |
| Hf-MOF | 2.0 | 0.40 | 4.4 | 0.56 | 29.2 | 0.04 | 3.0 |
| In-MOF | 0.7 | 0.60 | 2.6 | 0.37 | 12.5 | 0.03 | 1.4 |

1. **EXCITON DIFFUSION MODELING IN MOFs.**

The center-to-center distances and relative orientation between the DPA molecules in the MOF structures were determined using the structure of In-MOF solved from single-crystal X-ray diffraction analysis, and the crystal structures of Zr- and Hf-MOFs obtained from Rietveld refinements of PXRD patterns, as described in the literature. The center-to-center distances between pairs of DPA molecules were calculated from the centroid of the anthracene moieties within a radius of ~ 3 nm. The orientation of the anthracene moieties was defined by a vector along the shorter axis of the anthracene moiety, which is parallel to the transition electronic dipole vector calculated for the DPA molecule. The orientation between the different moieties was evaluated from the dihedral angle between the vectors of each DPA pair within a radius of ~ 3 nm.

The random diffusion of DPA singlet exciton within the Zr- and Hf-MOFs crystals has been modelled as hopping-mediated process occurring between isoenergetic centers, i.e. the framed DPA ligands. Dealing with a 3D isotropic structure, the singlet diffusivity can be calculated as

$D={k_{hop}^{DPA-DPA}a}^{2}$, Eq. S1

where $a$ = 1.2 nm is the center-to-center DPAs distance, $k_{hop}^{DPA-DPA}=\sum_{i} {m_{i}k}_{hop,i}^{DPA-DPA}$ is the total rate of the hopping process between a ligands pair and $m_{i}$ is the number of equivalent nearest neighbor sites surrounding the exciton at the distance *i*. Given that dipole permitted electronic transition involved and considering the short intermolecular distances involved, we can calculate the hopping ruled by Förster ET between DPA molecules, by neglecting exchange interaction–driven mechanism (Dexter ET). Therefore, with this assumption $k_{hop,i}$equals the Forster ET rate $k_{ET,i}^{DPA-DPA}$between two DPA, which can been calculated referring to the Förster radius $R_{fs}^{DPA-DPA}$. This latter is defined as the distance at which the ET efficiency is 0.5, and it can be derived from experimental data on the molar extinction coefficient $\varepsilon(\lambda)$ and photoluminescence $PL(\lambda)$ of DPA (Fig.S1). [1] Specifically, the radius value is given by

$R_{fs}^{DPA-DPA}=0.211\left[ J\left( \lambda\right)\theta^{2}n^{-4}\mathrm{QY}_{\mathrm{DPA}} \right]^{1/6}$, Eq. S2

where $\theta^{2}$ is the orientation factor between the transition momentum of the involved electronic transitions on the donor and acceptor systems, $n$ = 1.7 is the refraction index and $\mathrm{QY}_{\mathrm{DPA}}$= 0.96 is the DPA photoluminescence quantum yield. The overlap integral $J\left( \lambda\right)$is calculated form experimental data as

$J\left( \lambda\right)=\frac{\int PL(\lambda)\varepsilon(\lambda)\lambda^{4}d\lambda}{\int PL(\lambda)d\lambda}$ , Eq. S3

The DPA-DPA single pair ET rate is then calculated as

$k_{Fs}^{DPA/DPA}= {\frac{1}{\tau_{DPA}}\left( \frac{R_{fs}^{DPA-DPA}}{a_{i}} \right)}^{6}$ Eq. S4

where $\tau_{DPA}$ = 6.6 ns is the donor ligand lifetime in absence of the ligand acceptor (Fig. S11).

**Table S4.** Calculated values for $m_{i}$, $\theta^{2}$, $R_{FS}^{DPA/DPA}$, $k_{ET}^{DPA/DPA}$, $k_{hop}^{DPA/DPA}$ given every possible orientation of DPA molecules relative to one another with a distance cut-off at ca. 3.0 nm in the Zr- and Hf-MOFs.

| $a(Å)$ | $m_{i}$ | $\theta^{2}$ | $R_{fs}^{DPA/DPA}(Å)$ | $m_{i}k_{hop,i}^{DPA/DPA}(Hz)$ |
| --- | --- | --- | --- | --- |
| 11.62 | 8 | 1.55 | 26.28 | 1.62e+11 |
| 16.43 | 2 | 0.05 | 14.83 | 1.63e+8 |
| 16.43 | 4 | 2.25 | 27.96 | 1.47e+10 |
| 20.12 | 7 | 0.05 | 14.83 | 1.70e+7 |
| 20.12 | 7 | 3.05 | 29.42 | 1.03e+10 |
| 23.24 | 8 | 0.05 | 14.83 | 8.17e+7 |
| 23.24 | 2 | 1.05 | 24.63 | 4.29e+8 |
| 23.24 | 2 | 4.00 | 30.84 | 1.65e+10 |
| 25.98 | 16 | 0.05 | 14.83 | 8.37e+7 |
| 28.46 | 8 | 0.05 | 14.83 | 2.42e+7 |
| 30.74 | 14 | 0.05 | 14.83 | 2.67e+7 |
| 30.74 | 14 | 0.65 | 22.74 | 3.47e+8 |

The total hopping rate given by the Forster mechanism is calculated is $k_{hop}^{DPA-DPA}$= 190 GHz which corresponds to a diffusivity of *D_Fs_* = 2.6×10^-3^ cm^2^ s^-1^ which allows reaching the *rapid diffusion limit* regime when during scintillation a sufficiently larger density of excite singlets is generated. Notably, the real value of the hopping rate can be only larger than the one considered here since also exchange-mediated transfer and the contribution of the second/third nearest neighbors must be taken in to account, considering the close-packed arrangement of DPA ligands in the framework. All these effects would of course increase the diffusion speed of the exciton, thus further supporting the achievement of the rapid diffusion condition for the process with the proper density of singlet states present in the system.


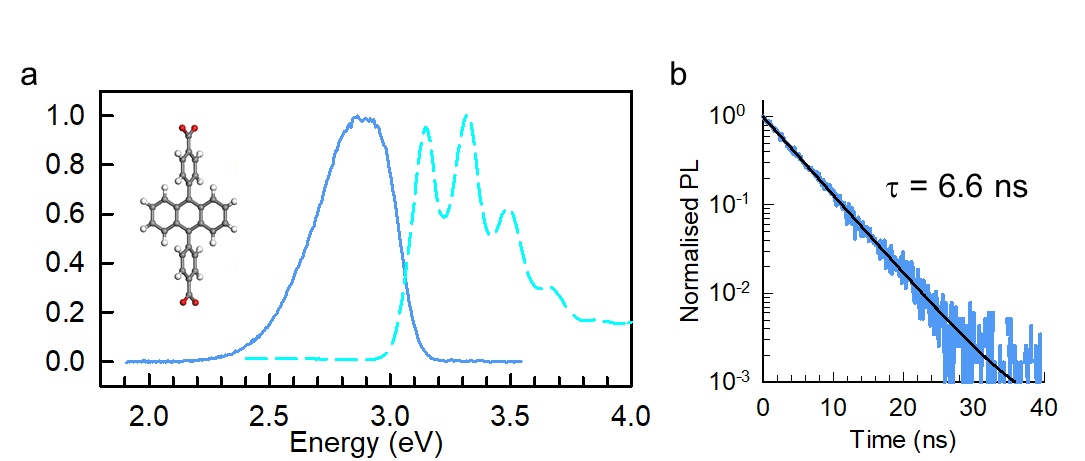


**Figure S11**. (a) Normalized absorption (dashed line) and photoluminescence (solid line) spectra of the ligand DPA in diluted THF solution under 340 cw excitation (10^-6^ M). The PL is peaked at 2.87 eV. (b) Fluorescence intensity decay at 2.87 eV recorded under pulsed laser excitation at 340 nm. Solid line is the fit of data with an exponential decay function of characteristic decay time τ = 6.6 ns.

Considering the In-MOF architecture, the total $k_{hop}^{DPA-DPA}$ rate requires a more complicated calculation due to the lower symmetry of the structure (Space group *Cmcm*). The unit cell contains two equivalent ligands (DPA-1 and DPA-2, accounting for 2/3 of the total rate) and one non-equivalent ligand (DPA-3, which counts for 1/3 of the total rate).

**Table S5.** Calculated values for $m_{i}$, $\theta^{2}$, $R_{FS}^{DPA/DPA}$, $k_{ET}^{DPA/DPA}$, $k_{hop}^{DPA/DPA}$ given every possible orientation of the equivalent DPA-1 and DPA-2 molecules in the In-MOF with a distance cut-off at ca. 3.0 nm.

| $a(Å)$ | $m_{i}$ | $\theta^{2}$ | $R_{fs}^{DPA/DPA}(Å)$ | $m_{i}k_{hop,i}^{DPA/DPA}(Hz)$ |
| --- | --- | --- | --- | --- |
| 7.28 | 2 | 0.024 | 13.12 | 1.04e+10 |
| 7.80 | 2 | 1 e-3 | 7.72 | 2.87 e+8 |
| 12.29 | 2 | 1.57 | 26.33 | 2.93e+10 |
| 13.21 | 2 | 1.73 | 26.77 | 2.10e+10 |
| 14.55 | 2 | 0.044 | 14.51 | 2.98e+8 |
| 14.55 | 2 | 0.75 | 23.27 | 5.08e+9 |
| 14.70 | 2 | 2.54 | 28.53 | 1.62e+10 |
| 15.60 | 2 | 0.28 | 19.82 | 1.28e+9 |
| 16.40 | 2 | 0.22 | 18.98 | 7.28e+8 |
| 16.40 | 2 | 0.45 | 21.42 | 1.50e+9 |
| 16.63 | 4 | 0.36 | 20.57 | 2.16e+9 |
| 18.84 | 2 | 0.34 | 20.41 | 4.90e+8 |
| 19.16 | 2 | 0.22 | 19.02 | 2.90e+8 |
| 19.16 | 2 | 3.27 | 29.76 | 4.26e+9 |
| 19.76 | 2 | 0.53 | 21.97 | 5.72e+8 |
| 19.76 | 2 | 3.59 | 30.22 | 3.87e+9 |
| 19.85 | 4 | 0.25 | 19.36 | 5.22e+8 |
| 20.43 | 2 | 0.58 | 22.30 | 5.12e+8 |
| 20.68 | 4 | 0.013 | 11.840 | 2.14e+7 |
| 20.68 | 4 | 0.42 | 21.11 | 6.86e+8 |
| 21.43 | 4 | 0.16 | 18.05 | 2.17e+8 |
| 21.83 | 2 | 0.024 | 13.12 | 1.43e+7 |
| 23.39 | 2 | 1 e-3 | 7.72 | 3.93e+5 |
| 23.89 | 2 | 0.60 | 22.43 | 2.08e+8 |
| 23.89 | 2 | 0.68 | 22.90 | 2.35e+5 |
| 24.57 | 2 | 0.060 | 15.28 | 1.76e+7 |
| 24.7 | 4 | 0.39 | 20.92 | 2.23e+8 |
| 24.7 | 4 | 0.55 | 22.14 | 3.14e+8 |
| 25.17 | 2 | 0.74 | 23.25 | 1.88e+9 |
| 25.17 | 2 | 0.86 | 23.83 | 2.18e+8 |
| 25.78 | 4 | 1.30 | 25.521 | 5.70e+8 |
| 26.31 | 2 | 0.09 | 16.44 | 1.80e+7 |
| 26.31 | 2 | 0.30 | 19.96 | 5.78e+7 |
| 26.42 | 2 | 1e-3 | 7.72 | 1.89e+5 |
| 26.42 | 2 | 0.92 | 24.10 | 1.74e+8 |
| 26.82 | 2 | 1.47 | 26.04 | 2.53e+8 |
| 27.62 | 4 | 0.27 | 19.64 | 7.83e+7 |
| 27.97 | 2 | 1.58 | 26.35 | 2.12e+8 |
| 28.63 | 4 | 0.075 | 15.86 | 1.75e+7 |
| 29.10 | 2 | 0.044 | 14.51 | 4.66e+6 |
| 29.10 | 2 | 0.75 | 23.27 | 7.93e+7 |
| 29.39 | 2 | 0.62 | 22.47 | 6.05e+7 |
| 29.68 | 4 | 0.51 | 21.87 | 9.71e+7 |
| 29.68 | 4 | 1.40 | 25.84 | 2.64e+8 |

**Table S6.** Calculated values for $m_{i}$, $\theta^{2}$, $R_{FS}^{DPA/DPA}$, $k_{ET}^{DPA/DPA}$, $k_{hop}^{DPA/DPA}$ given every possible orientation of the equivalent DPA-3 molecules in the In-MOF with a distance cut-off at ca. 3.0 nm.

| $a(Å)$ | $m_{i}$ | $\theta^{2}$ | $R_{fs}^{DPA/DPA}(Å)$ | ${m_{i}k}_{hop,i}^{DPA/DPA}(Hz)$ |
| --- | --- | --- | --- | --- |
| 7.28 | 4 | 0.024 | 13.12 | 2.08e+10 |
| 13.21 | 8 | 1.73 | 26.77 | 8.40e+10 |
| 14.55 | 4 | 0.45 | 21.41 | 6.161e+9 |
| 14.70 | 2 | 2.63 | 28.69 | 1.68e+10 |
| 15.59 | 2 | 0.018 | 12.50 | 8.07e+7 |
| 16.40 | 4 | 0.45 | 21.42 | 3.01e+9 |
| 16.40 | 4 | 0.22 | 18.98 | 1.46e+9 |
| 18.84 | 4 | 0.34 | 20.41 | 9.80e+8 |
| 19.76 | 4 | 0.53 | 21.97 | 1.14.e+9 |
| 19.76 | 4 | 3.50 | 30.22 | 7.75e+9 |
| 20.43 | 4 | 0.58 | 22.30 | 1.024e+9 |
| 20.68 | 8 | 0.29 | 19.86 | 9.51e+8 |
| 21.43 | 4 | 1e-3 | 7.72 | 1.33e+6 |
| 21.83 | 4 | 0.024 | 13.12 | 2.86e+7 |
| 23.89 | 4 | 0.60 | 22.43 | 4.15e+8 |
| 23.89 | 4 | 0.68 | 22.90 | 4.70e+8 |
| 24.57 | 2 | 1 | 24.43 | 2.92e+8 |
| 25.17 | 4 | 0.744 | 23.25 | 3.77e+8 |
| 25.17 | 4 | 0.86 | 23.83 | 4.36e+8 |
| 26.31 | 4 | 0.09 | 16.44 | 3.61e+7 |
| 26.31 | 4 | 0.30 | 19.96 | 1.16e+8 |
| 26.42 | 4 | 0.012 | 11.69 | 4.55e+6 |
| 26.82 | 4 | 1.47 | 26.038 | 5.07e+8 |
| 27.97 | 4 | 1.58 | 26.35 | 4.24e+8 |
| 28.63 | 4 | 0.062 | 15.37 | 1.45e+8 |
| 29.10 | 4 | 0.45 | 21.417 | 9.63e+7 |
| 29.40 | 2 | 0.75 | 23.27 | 7.45e+7 |

The total hopping rate given by the Forster mechanism in in In-MOF is $k_{hop}^{DPA-DPA}$= 112 GHz which corresponds to a Förster mediated diffusivity of *D_Fs_* = 6.23×10^-4^ cm^2^ s^-1^. Importantly, also in this MOF the real hopping rate is larger than the calculated only considering Forster interactions, especially in this case where the significantly more compact structure put the first neighbor ligands at distance much short than 1 nm with respect to the Zr- and Hf-MOFs, thus strongly favoring exciton coupling through short range interactions as in the Dexter case.

- 1. **Estimation of Dexter transfer contribution to hopping**

The homo-molecular Dexter energy transfer contribution to the singlet excitons hopping rate in In-MOFs has been estimated from time resolved photoluminescence and scintillation data combined with the result obtained on the modeling of the homo-molecular Förster energy transfer discussed above.

**Table S7.** Luminescence intensity decay rates under UV or X-rays pulsed excitation for the investigated MOF series. The rates values has been calculated from the experimental characteristic decay time data as $k_{i}=\left( \tau_{i} \right)^{-1}$.

| sample | $k_{pl}$ (GHz) | $k_{scint}$ (GHz) | $k_{SSA}=k_{scint}$ -$k_{pl}$ (GHz) |
| --- | --- | --- | --- |
| Zr-MOF | 0.33 | 0.37 | 0.04 |
| Hf-MOF | 0.33 | 0.50 | 0.17 |
| In-MOF | 0.71 | 1.11 | 0.40 |

As already demonstrated (Refs. 52, 57) the singlet-singlet annihilation rate in occurring in MOFs can be calculated in the rapid diffusion limit as

$k_{\mathrm{SSA}}=8\pi DR_{\mathrm{SSA}}[S^{*}]$ , Eq.S5

where $R_{\mathrm{SSA}}$ is the interaction distance between two DPA singlets, $[S^{*}]$ is the density of excited diffusing singlets and $D$ is the exciton diffusivity by homo-molecular hopping mediated by the Förster and Dexter mechanisms. Specifically, the diffusivity in the MOF crystalline framework can be calculated as reported in Eq. S1

$D={k_{hop}^{DPA-DPA}a}^{2}= \left( k_{Fs}^{DPA-DPA}+k_{Dx}^{DPA-DPA} \right)a^{2}$, Eq. S6

where $k_{Fs}^{DPA-DPA}$and $k_{Dx}^{DPA-DPA}$ are the Forster and Dexter contribution, respectively, and $a$ is the DPA-DPA center to center distance between two ligands. In first approximation, it takes the value of 11.8 Å and 7.5 Å for Zr- and Hf-MOFs and In-MOF, respectively. From the data reported in Table S4, we can therefore estimate the ratio between two different exciton diffusivities as

$\frac{D_{MOF-1}}{D_{MOF-2}}=\frac{\left( {k_{hop}^{DPA-DPA}a}^{2} \right)_{MOF-1}}{\left( {k_{hop}^{DPA-DPA}a}^{2} \right)_{MOF-2}}=\frac{\left( k_{\mathrm{SSA}} \right)_{MOF-1}}{\left( k_{\mathrm{SSA}} \right)_{MOF-2}}$. Eq. S7

We can therefor use Eq. S7, to evaluate the Dexter contribution to the singlets diffusivity in the In-MOF by comparing the $k_{\mathrm{SSA}}$ rate with that one of the Zr-MOF, which shows a similar Z number. In this way, as discussed in the main text, we can consider the same density of excited states generated in the MOFs upon interaction with x-rays. Moreover, we know from previous investigations that the Dexter contribution to the singlet diffusivity in the Zr- and Hf-MOFs is negligible (Refs. 52), in agreement with the spatial arrangement of ligands that quite well separate their conjugated cores minimizing the short-range exchange interaction that rules the Dexter mechanism (Refs 74, 75). By substituting experimental data from Table S4 in Eq. S5 and considering the $k_{Fs}^{DPA-DPA}$ values calculated for the different MOFs as discussed above, from Eq. S7 we obtain for the In-MOF a $k_{Dx}^{DPA-DPA}$ as large as 4.59 THz.

1. **additional LUMINESCENCE DATA.**

**Figure S12**. RL spectrum of the In-MOF as synthesized and after 10 months of storage in open air.


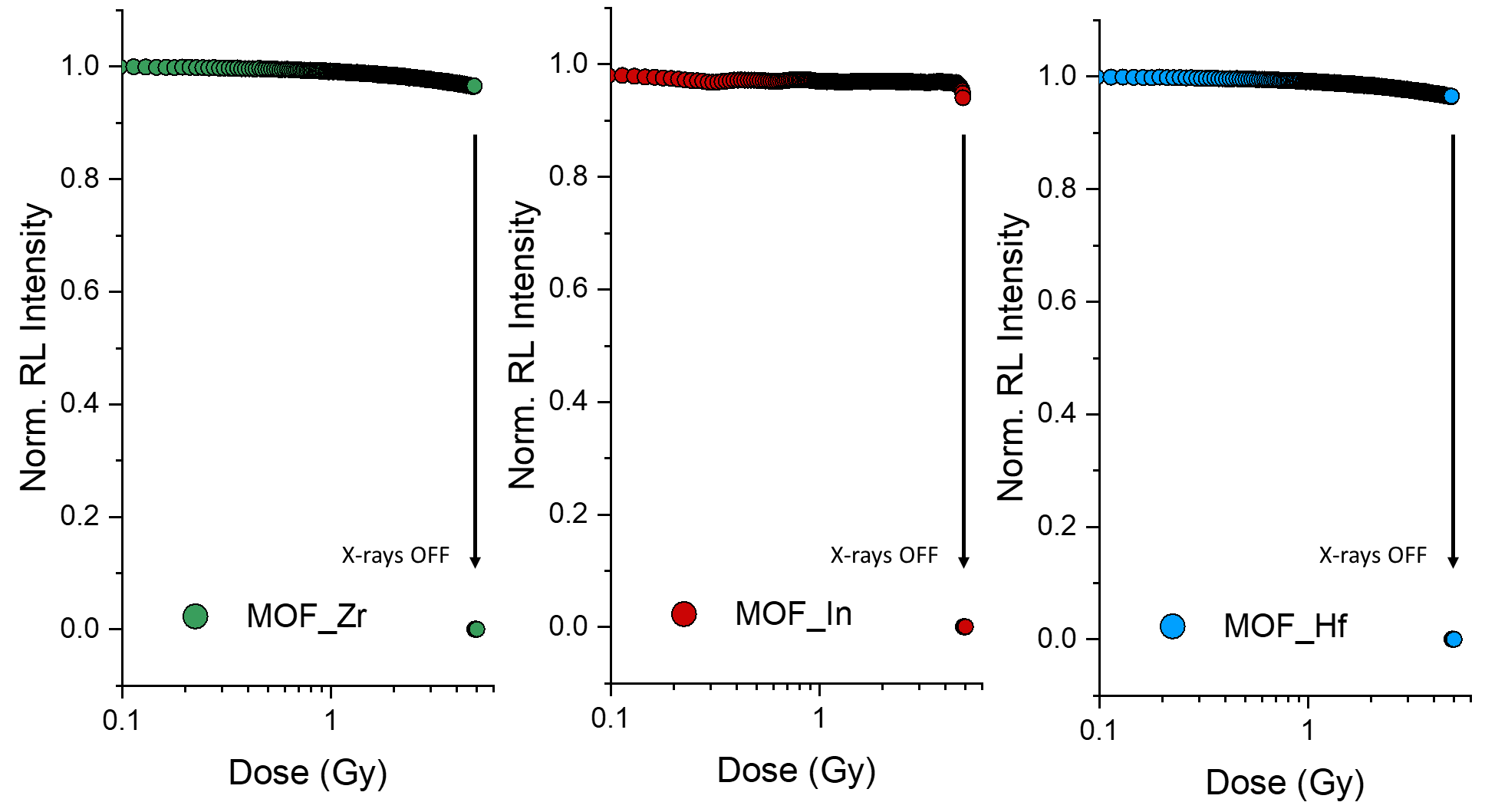


**Figure S13**. Integrated RL amplitude measured as a function of the delivered dose. The employed dose rate is 5 mGy/s.

**
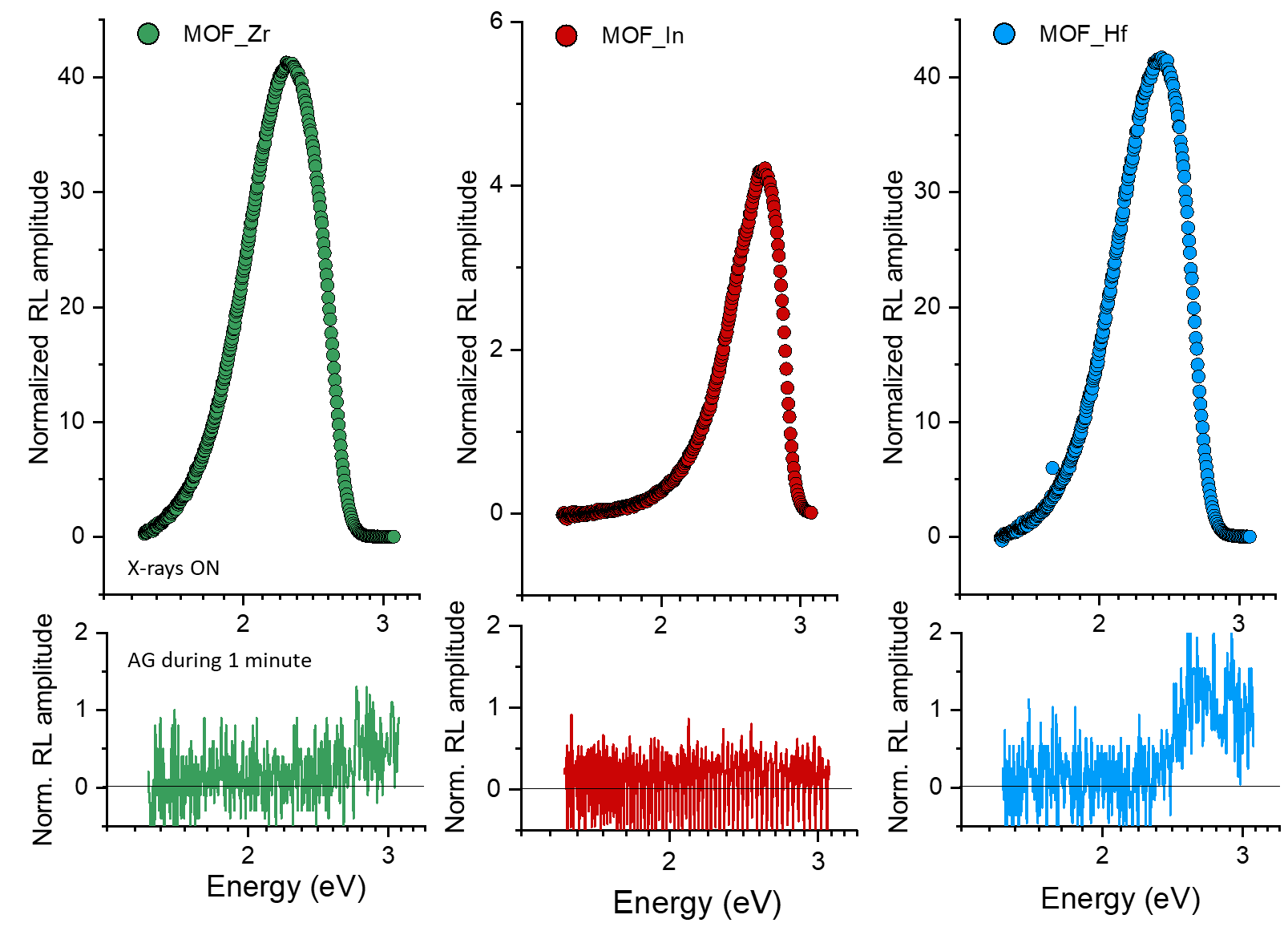
**

**Figure S14**. Comparison between the intensity of the RL of MOF under X-rays exposure (top panels) and of the afterglow emission integrated for 1 minute after switching off the X-rays excitation (bottom panels).


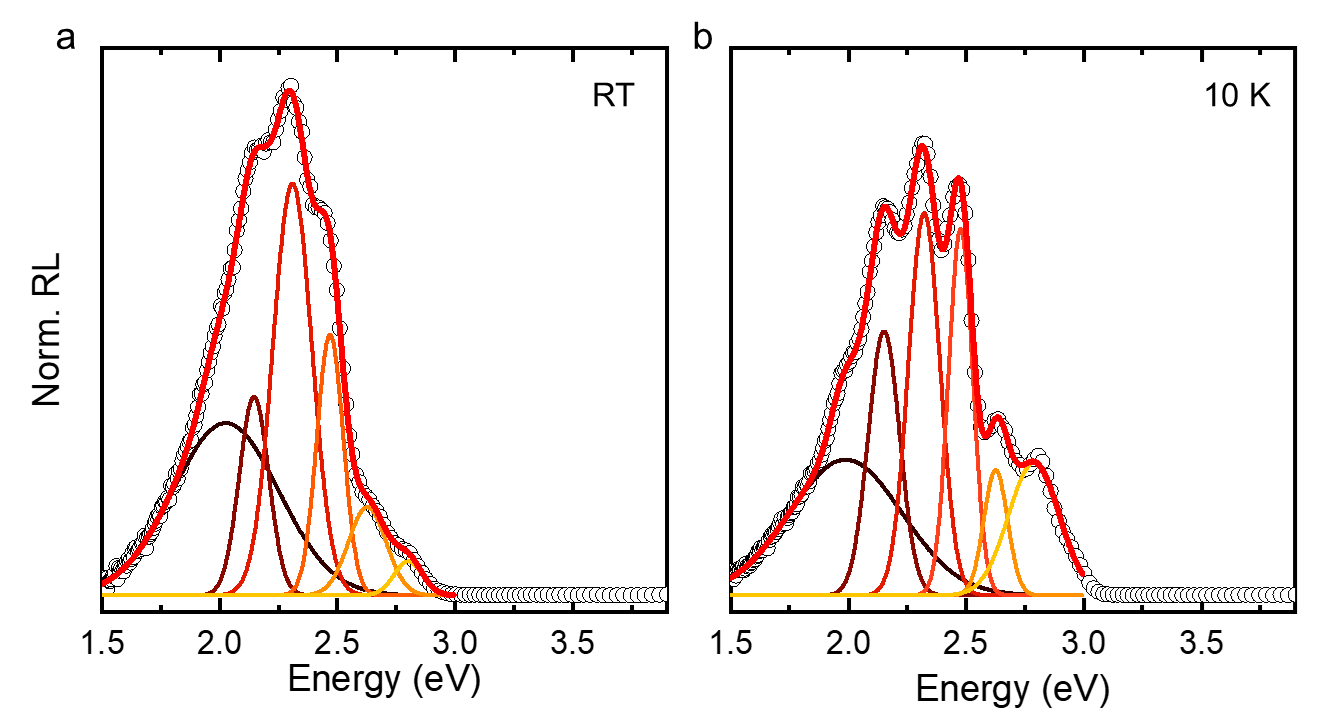


**Figure S15**. Normalized radioluminescence spectra of the conjugated ligand DPA (open symbols) recorded under soft X-rays excitation at RT (a) and 10 K (b). We report the global fit of the emission spectra with a sum of six Gaussian contributions (solid lines).


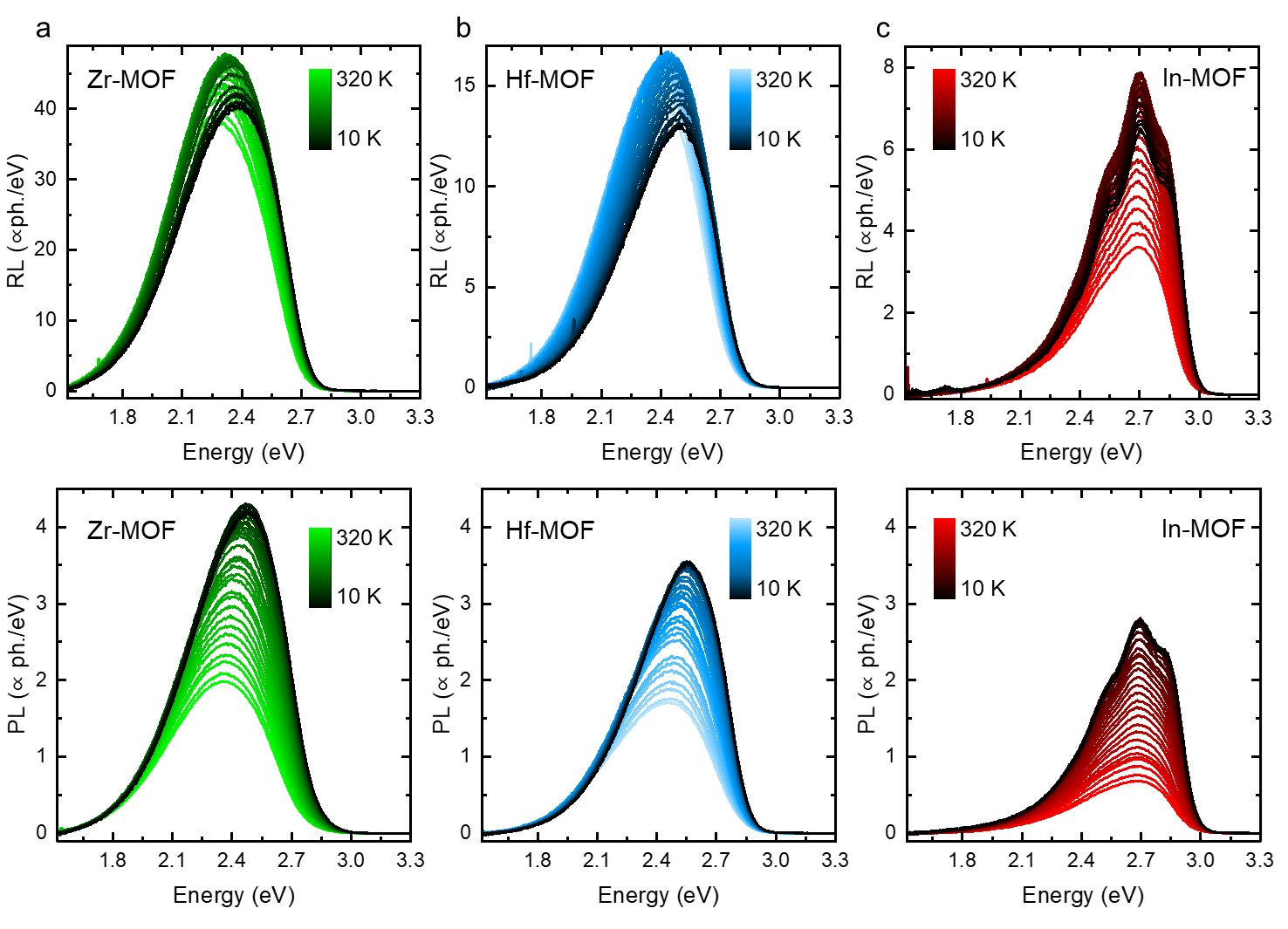


**Figure S16**. RL and PL spectra as a function of temperature for Zr-MOF (a), Hf-MOF (b), and In-MOF (c) under soft X-ray or laser excitation at 3.5 eV (266 nm).


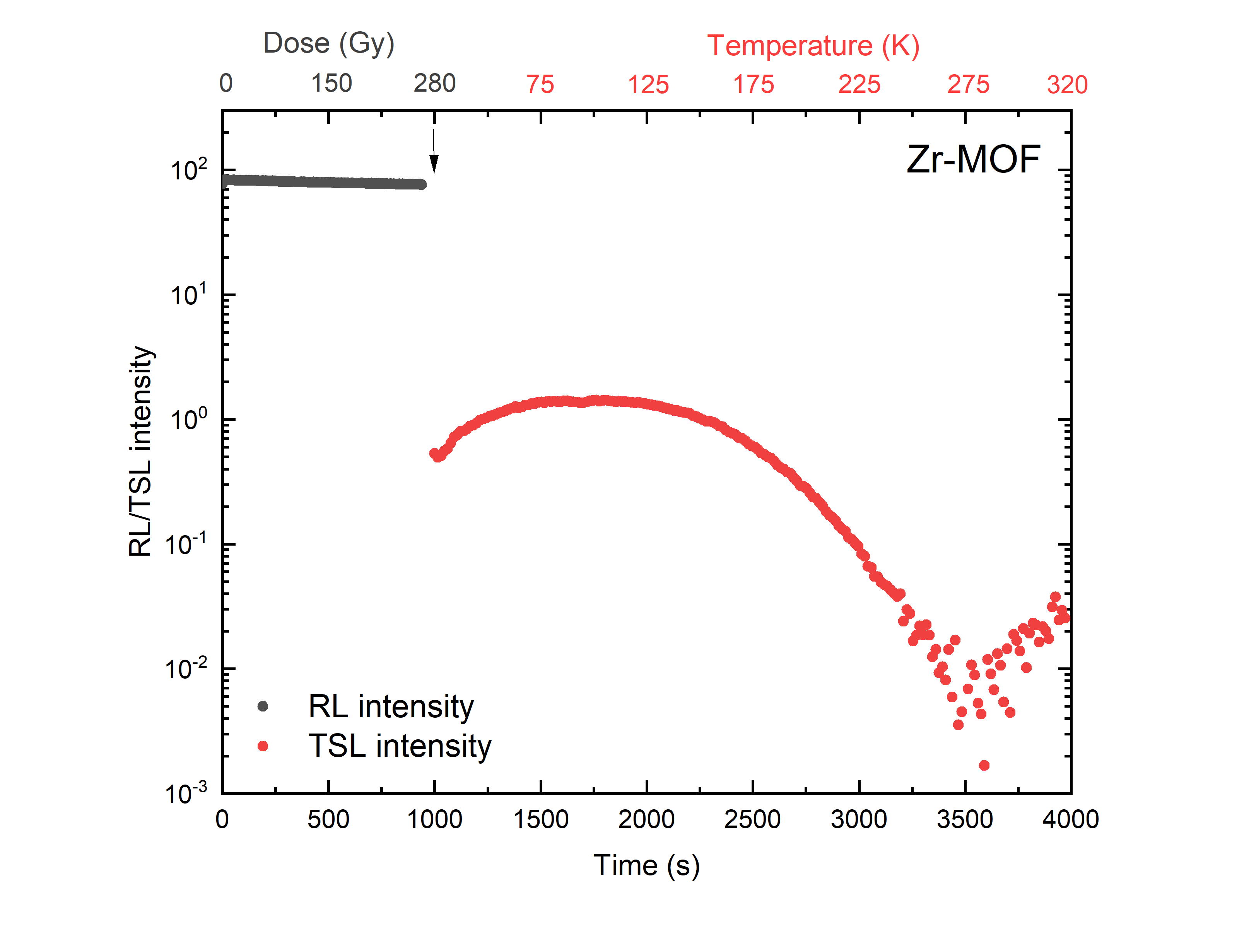

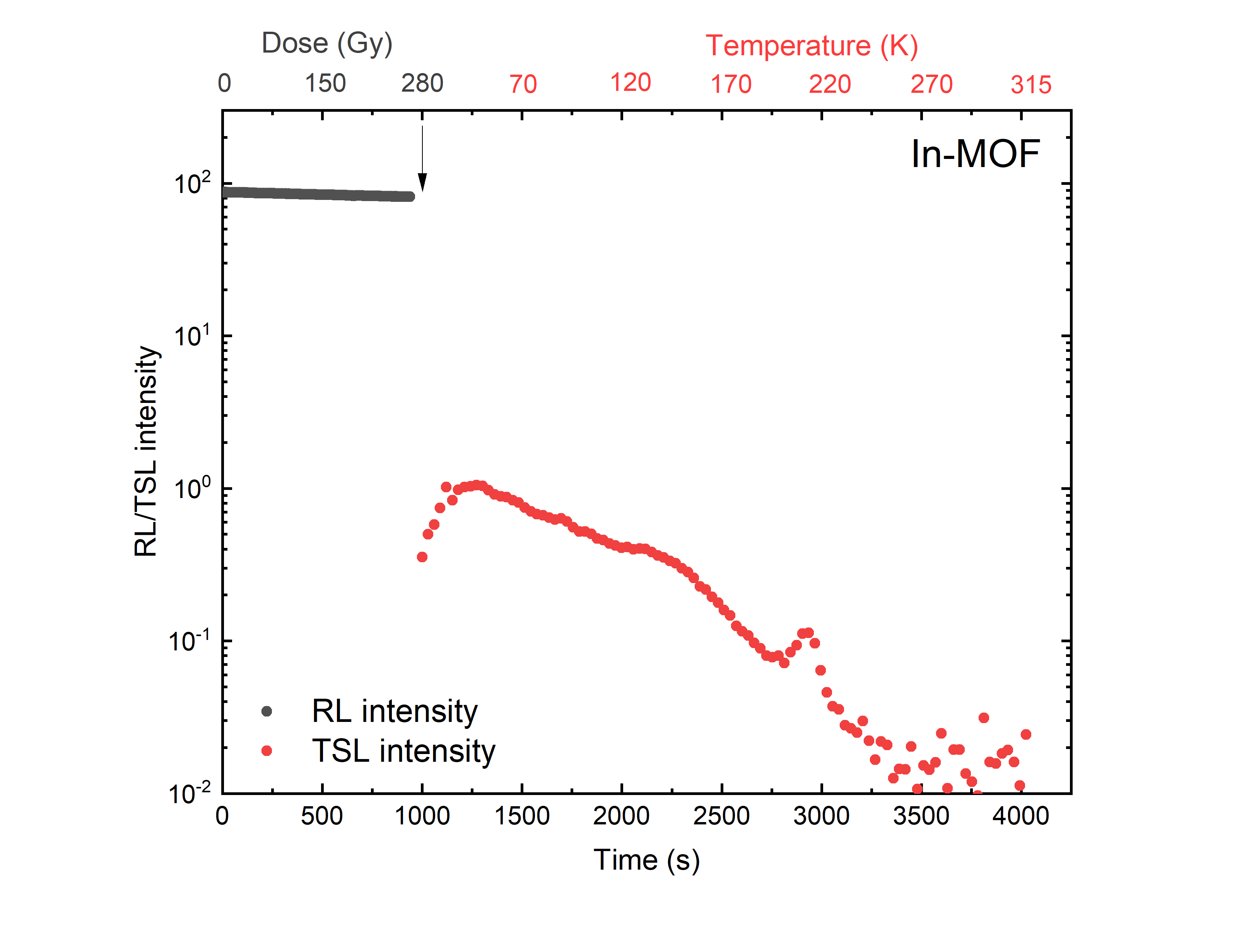


X-rays ON

X-rays ON

**Figure S17**. We make a qualitative evaluation of the influence of defects in the scintillation performance of MOF nanocrystals by their RL and TSL response. We acquired the radioluminescence signal at 10 K emitted during the irradiation preceding the TSL measurement as a function of cumulated dose (proportional to the exposure time since a constant dose rate of 5 mGy/s was used): the integral of the radioluminescence emission over dose (𝐼_𝑅𝐿_) is due to the prompt recombination of free carriers generated by irradiation up to a total X-ray dose of around 5 Gy. Conversely, the integral of the glow curve (𝐼_𝑇𝑆𝐿_), acquired after the aforementioned irradiation at 10 K, is related to the delayed recombination of previously trapped charge carriers thermally released upon heating. We could estimate the amount of light originating from the thermal release of trapped carriers and not contributing to the prompt scintillation response by calculating

$$T_{\%}=\frac{I_{TSL}}{I_{RL}+I_{TSL}}$$

where 𝐼_𝑇𝑆𝐿_ and 𝐼_𝑅𝐿_ have been previously defined. Our approach allows to evaluate in an operative way the contribution of trapping phenomena with respect to prompt scintillation. We directly compare the 𝑇_%_ of our systems and obtain 𝑇_%_=2.4 % for Zr-based MOF nanocrystals, 𝑇_%_=1.2 % for In-MOF, and T_%_= 2.5% for Hf-MOF as reported by M. Orfano et al. (Ref. 55).


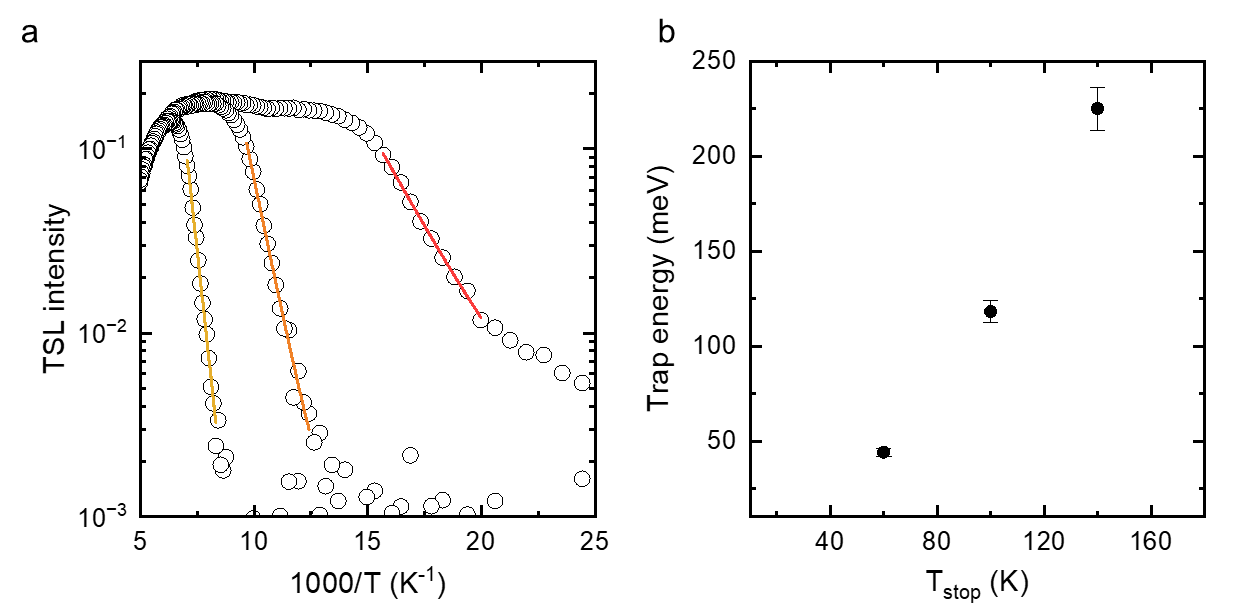


**Figure S18**. (a) Arrhenius plot of the TSL measurements obtained with the partial cleaning method: the rising portion of the resulting glow curve is fitted with a single exponential function to evaluate the trap energy depth. (b) Distribution of trap energy values calculated by the initial rise method versus T_stop_.

**Supplementary References**

[1] Lakowicz, J. R. Principles of fluorescence spectroscopy. (Springer Science+Business Media, 2010).

[2] Stryer, L., Thomas, D. D. & Meares, C. F. Diffusion-enhanced fluorescence energy transfer. Annual review of biophysics and bioengineering 11, 203-222 (1982).
